# Biomining *Sesuvium portulacastrum* for halotolerant PGPR and endophytes for promotion of salt tolerance in *Vigna mungo* L.

**DOI:** 10.3389/fmicb.2023.1085787

**Published:** 2023-02-14

**Authors:** Joseph Ezra John, Muthunalliappan Maheswari, Thangavel Kalaiselvi, Mohan Prasanthrajan, Chidamparam Poornachandhra, Srirangarayan Subramanian Rakesh, Boopathi Gopalakrishnan, Veeraswamy Davamani, Eswaran Kokiladevi, Sellappan Ranjith

**Affiliations:** ^1^Department of Environmental Sciences, AC&RI, Tamil Nadu Agricultural University, Coimbatore, India; ^2^Department of Agricultural Microbiology, AC&RI, Tamil Nadu Agricultural University, Coimbatore, India; ^3^ICAR-National Institute of Abiotic Stress Management, Baramati, India; ^4^Department of Biotechnology, Agricultural College and Research Institute, Tamil Nadu Agricultural University, Coimbatore, India

**Keywords:** Halophyte, rhizobacteria, endophytes, salt tolerance, crop improvement, ACC deaminase

## Abstract

Halophytic plants can tolerate a high level of salinity through several morphological and physiological adaptations along with the presence of salt tolerant rhizo-microbiome. These microbes release phytohormones which aid in alleviating salinity stress and improve nutrient availability. The isolation and identification of such halophilic PGPRs can be useful in developing bio-inoculants for improving the salt tolerance and productivity of non-halophytic plants under saline conditions. In this study, salt-tolerant bacteria with multiple plant growth promoting characteristics were isolated from the rhizosphere of a predominant halophyte, *Sesuvium portulacastrum* grown in the coastal and paper mill effluent irrigated soils. Among the isolates, nine halotolerant rhizobacterial strains that were able to grow profusely at a salinity level of 5% NaCl were screened. These isolates were found to have multiple plant growth promoting (PGP) traits, especially 1-aminocyclopropane-1-carboxylic acid deaminase activity (0.32–1.18  μM of α-ketobutyrate released mg^−1^ of protein h^−1^) and indole acetic acid (9.4–22.8  μg mL^−1^). The halotolerant PGPR inoculation had the potential to improve salt tolerance in *Vigna mungo* L. which was reflected in significantly (*p* < 0.05) higher germination percentage (89%) compared to un-inoculated seeds (65%) under 2% NaCl. Similarly, shoot length (8.9–14.6 cm) and vigor index (792–1785) were also higher in inoculated seeds. The strains compatible with each other were used for the preparation of two bioformulations and these microbial consortia were tested for their efficacy in salt stress alleviation of *Vigna mungo* L. under pot study. The inoculation improved the photosynthetic rate (12%), chlorophyll content (22%), shoot length (5.7%) and grain yield (33%) in *Vigna mungo* L. The enzymatic activity of catalase and superoxide dismutase were found to be lower (7.0 and 1.5%, respectively) in inoculated plants. These results revealed that halotolerant PGPR isolated from *S. portulacastrum* can be a cost-effective and ecologically sustainable method to improve crop productivity under high saline conditions.

## 1. Introduction

Agricultural productivity is very important to ensure food security in future with the ensuing population rise. As per the Global Agricultural Productivity (GAP) Index, the current growth rate of agricultural production is not enough to meet the projected food demand of 10 billion people in 2050 (Zeigler and Steensland, 2022). In addition to this, plants are confronted with various kinds of stresses such as drought, flooding, salinity, heat, cold, nutrient deficiency and exposure to heavy metals, phytopathogen, pest attack, etc. Among various environmental stressors, excessive presence of salts in soil (soil salinity) is one of the major problems responsible for the reduction of crop growth and productivity across the globe. It is reported that more than 1,000 million hectares of land are affected by salinity throughout the world (Negacz et al., 2022). Globally, about 10% of agricultural soils are under the threat of salinization due to continuous usage of fertilizer and poor-quality water for irrigation (Borsato et al., 2020; Kumar and Sharma, 2020). Salinity adversely affects crop productivity in arid and semi-arid areas around the world where it causes an annual loss of 1–2% of arable land (Shrivastava and Kumar, 2015). Soil salinity also induces biochemical changes in the salt sensitive crop owing to disturbance in osmotic potential, imbalance in ion concentration, and increased Reactive Oxygen Species (ROS) production (Singh et al., 2022). This leads to the break in electron transport chain and cleavage of hydrogen bond between the amino acids in the genetic material which has deleterious effect on crop (Ermakova et al., 2019). Furthermore, high concentration of salts in the plant cells were reported to induce oxidative stress and enhanced production of stress ethylene, which in turn affects the physiological processes like respiration, photosynthesis, nitrogen fixation, etc. (Gupta and Huang, 2014; Paul and Lade, 2014; Acosta-Motos et al., 2017).

Removal of salt from saline soil is an intensive process which is time consuming and requires financial investment (Qadir et al., 2014). However, for a long time, the reclamation of saline soils was carried out mainly by physical and chemical processes. In physical process, soluble salts in the root zone are removed by scraping, flushing and leaching methods, while the use of gypsum and lime as neutralizing agents in saline soils is employed in chemical method (Ayyam et al., 2019). But these methods are not sustainable when the salt concentration is too high in soils continuously irrigated with saline water. Unless these salts are leached from the soil, they accumulate to levels that are inhibitory to plant growth and may lead to soil salinity. In the long run, salinity causes the degradation of soil structure affecting water and root penetration along with other problems (Gul et al., 2014). In these cases, phytoremediation comes as a viable alternative to ensure soil productivity, preferably halophytes that have evolved to grow in saline soils and uptake salt (Flowers and Colmer, 2015). With considerable progress in understanding physiological and molecular mechanisms in salt tolerance of halophytes, some have been found to possess genes suitable for improving salt tolerance and phytoremediation potential (Shabala, 2013; Gul et al., 2014; Diray-Arce et al., 2015). Besides this, microbes associated with rhizosphere are known to promote the desired effect like, growth regulation, remediation potential, biotic and abiotic stress tolerance, etc. (Upadhyay et al., 2022a). Microbes found in the rhizosphere (rhizobacteria) or within plant tissues including roots (endophytes) have the potential to contribute significantly to the ability of plants to adapt to adverse conditions (Numan et al., 2018; Chauhan et al., 2022). However, the potential contribution of microorganisms associated with these plants in the soil, on plant surfaces, or within plant tissues is underutilized.

Exogenous compounds produced by microorganisms in the rhizosphere region promote nutrient uptake, control pathogens, and lessen the effects of salinity and sodicity (Damodaran et al., 2013). These include Indole Acetic Acid (IAA) production (Ahemad and Kibret, 2014), Hydrogen Cyanide (HCN) production, siderophore production, 1-Aminocyclopropane-1-carboxylate (ACC) deaminase production, nutrient solubilization and suppression of soil borne pathogens (Dimkpa et al., 2009). Furthermore, certain microorganisms promote the activity of plant anti-oxidants and osmolytes production (Etesami and Beattie, 2018). The direct and indirect mechanisms, metabolism and chemotaxis in promotion of growth in plants are interceded by gene cluster that activates host–PGPR interactions. In *Bacillus subtilis*-GB03, out of 38 genes, 30 genes responded to the change in the root structure of the host (*Arabidopsis* sp.) in addition to growth promotion (Ryu et al., 2003). The upsurge in nutrient availability and production of plant growth regulators (IAA, ACC deaminase, ethylene and gibberellic acid) are direct mechanisms through which microbes improve crop growth (Upadhyay et al., 2022b). The siderophore production is known to promote iron availability that has direct impact on crop growth. Siderophores also exert antimicrobial activity by limiting iron availability to the pathogens. The EPS, HCN and hydrolytic enzyme production has various indirect benefits to the crop such as antipathogenic potential, disease resistance and abiotic stress tolerance (Upadhyay et al., 2011, 2022b). Microbial isolates with PGP characteristics from rhizosphere regions of halophytes like *A. nummularia* (Da Silva et al., 2016) and *Salicornia* sp. (Mapelli et al., 2013) could provide an alternative option for chemical amendments. Characterizing these bacteria from saline environments may lead to the identification of beneficial microorganisms for use as inoculants to stimulate the growth of non-host plants under saline conditions.

In an earlier study with the halophyte *Sesuvium portulacastrum* collected from the coastal soils of Tamil Nadu, India, it was observed that *S. portulacastrum* had the potential to mitigate salination of soil irrigated with paper and pulp mill effluent containing high salt (John et al., 2022). This experiment was conducted in paper and pulp mill effluent irrigated area near Tamil Nadu Newsprint and Papers Limited (TNPL), Karur, Tamil Nadu, India and it is known as the Treated Effluent Water Lift Irrigation System area (TEWLIS). The current practice for managing saline soils among the farming community is flooding and leaching of salts with good quality water. However, the lack of good quality water, expertise in soil drainage and high-cost requirements discourage them from practicing it especially, where wastewater is the only source for irrigation. In this case, microbial assisted agriculture could improve the crop growth and yield which requires minimum skill and cost. Halophytes harbor salt tolerant microorganisms as endophytes and epiphytes which are also capable of growing in saline environment. These microorganisms have been reported to enhance biotic (Masum et al., 2018) and abiotic stress (Upadhyay et al., 2011) tolerance in many crops. However, endophytes of *Sesuvium portulacastrum* have not been reported yet. Due to its ability to grow in higher concentration of salt, it is felt that the culturable endo and epiphytic bacteria of *Sesuvium portulacastrum* could tolerate salinity and also trigger induced systemic tolerance against salinity stress in plant. Keeping this in view, the current study was conceived to isolate epiphytic and endophytic plant growth promoting bacterial flora from halophyte, *S. portulacastrum* grown in salt affected ecosystem. Further investigation was done to validate the potential of halotolerant plant growth promoting bacterial effects on salt sensitive crop (*Vigna mungo* L.) grown in saline condition. A total of 31 isolates were obtained from rhizosphere of *S. portulacastrum* of which nine strains were found to be halotolerant. These isolates were evaluated for their PGP characteristics, tested under *in vitro* for their growth promotion in *Vigna mungo* L. at 2% NaCl and were identified through 16 s RNA sequencing. All the nine isolates had at least one PGP capability, hence two consortia with three strains in each were formulated based on the compatibility between the isolates. Microbial consortium I (MC I) had *Metabacillus indicus, Neobacillus niacini* and *Serratia marcescens*; while MC II (Microbial consortium II) was formulated with *Bacillus velezensis, Kocuria rhizophila*, and *Kosakonia radicincitans*. Consequently, their potential in growth promotion of salt sensitive glycophyte, *Vigno mungo* L. in paper and pulp mill effluent irrigated soil under pot culture experiment was confirmed.

## 2. Materials and methods

### 2.1. Collection of samples

**Figure 1:**
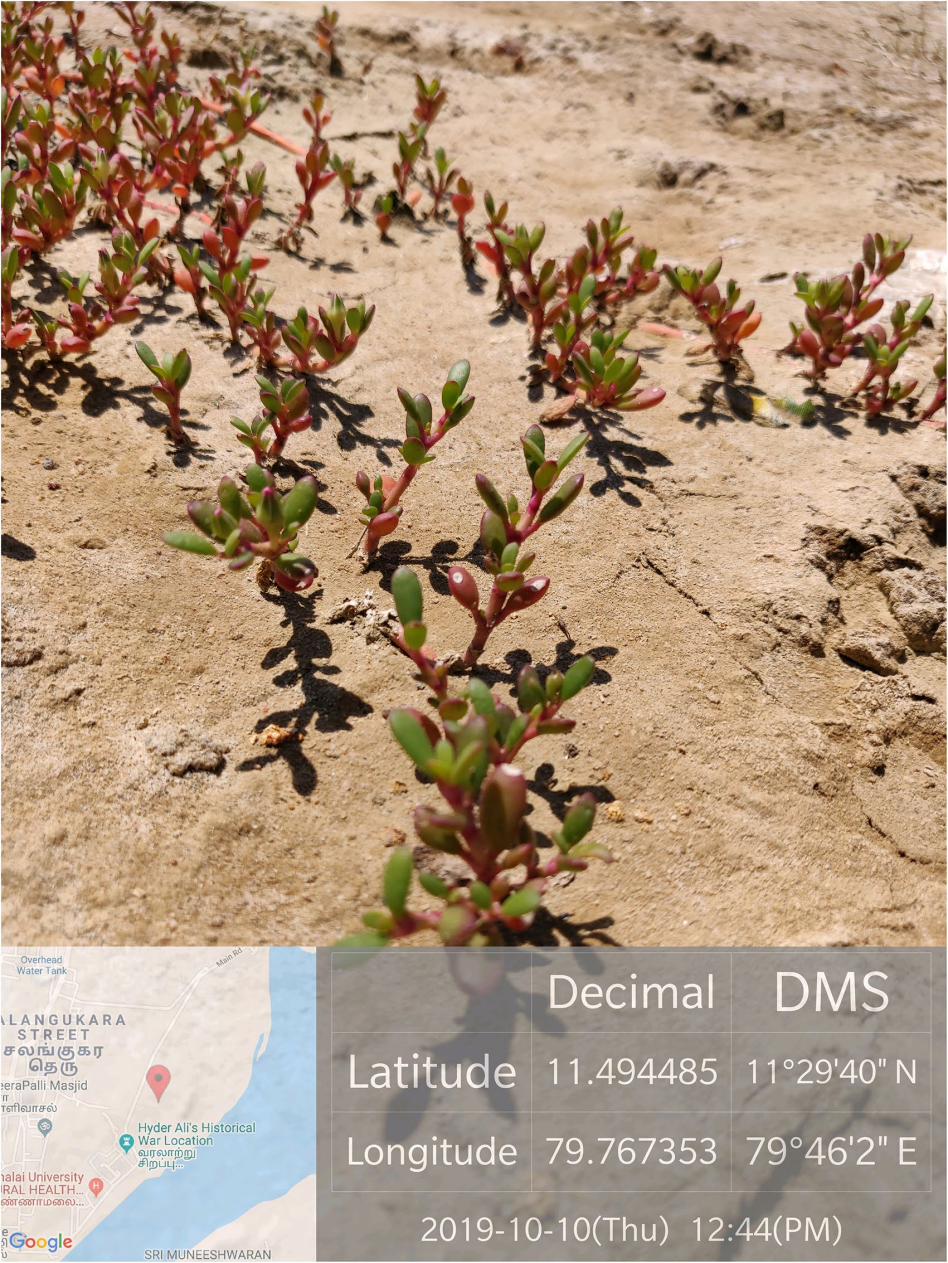
Collection of *Sesuvium portulacastrum* from the shores of backwaters at Parangipettai, Tamil Nadu, India.

The soil and plant samples were initially collected from the coastal area of Parangipettai, Tamil Nadu, India. The *S. portulacastrum* was mostly found on the sandy shores of the backwaters (Figure 1). The collected halophyte was successfully established in the soil salinized by paper and pulp mill effluent irrigation at Karur, Tamil Nadu, India. The rhizospheric soil from two soil series, Thulukanur and Vannapatti soil series in the paper mill effluent irrigated area was collected. The samples were collected in sterile plastic bags and transferred to laboratory for further analysis. The organic matter was analyzed by the Walkley-Black method while Electrical Conductivity (EC) and pH were analyzed by the saturated paste extract method (Murtaza et al., 2017). The physico chemical properties are tabulated in Supplementary Table S3. Soil samples required for the study were collected from the soils of long-term treated paper and pulp mill effluent irrigated area located at 11° 01′24.9″ N and 77° 59′59″ E. Adequate amount of soil was shade dried, large debris was removed and subsequently 10 kg of soil was transferred to perforated pots for secondary evaluation. The pH and EC of the experimental soil were found to be 8.18 and 2.62 dS m^−1^, respectively (Supplementary Table S3). The Exchangeable Sodium Percentage (ESP) of the soil was 13.54 per cent with an organic carbon content of 0.63 per cent.

### 2.2. Isolation and characterization of the halotolerant bacteria

To isolate rhizospheric bacteria, plant roots were gently shaken to remove the clumps of loosely adhering soil to the roots, leaving behind the root-firmly adhering soil particles (rhizospheric soil), which were then suspended and vortexed in 100 mL of sterile 1% NaCl solution. Thereafter, 10 folded serial dilutions (10×, 20×, 30×, 40×, and 50×) were prepared and 1 ml from each diluent was plated on nutrient agar (NA) medium (Supplementary Table S1) supplemented with 2% NaCl. The plates were incubated at 28°C and monitored for colony formation for up to 1 week (Fisher et al., 2007). For the isolation of endophytic bacteria, the roots of the collected samples were washed carefully under running tap water for 10 min to remove adhering soil particles. The roots were disinfected with 70% ethanol for 1 min, then rinsed three times with sterile distilled water. The roots were then surface sterilized with 3% sodium hypochlorite solution containing a few drops of Tween 20 R (Sigma-Aldrich, Steinheim, Germany) for 10 min followed by six rinses with sterile distilled water. To confirm root surface sterilization efficiency, an aliquot (100 mL) from the sixth wash solution was spread on NA medium and incubated at 28°C for 5 days. Thereafter, 1 g of the surface sterilized root tissue was macerated with a sterilized mortar and pestle in 10 mL distilled water and 1 mL from the tissue extracts and the serial diluents (10×, 20×, and 30×) were spread on NA medium supplemented with 2% NaCl. The plates were incubated at 28°C and monitored for up to 1 week for bacterial colony formation (Ramadoss et al., 2013).

The colonies of rhizospheric and endophytic bacterial isolates were examined morphologically for their shape, size, margin, elevation, appearance, texture, and pigmentation. In addition, cellular morphology, shape, Gram staining and biochemical characters were also examined (Sandhya and Ali, 2018; Supplementary Table S2). Colonies with distinct morphological characteristics were selected and purified by subculturing three times on NA media supplemented with 3% NaCl, before their storage in a 40% glycerol solution at −20°C till further use. Each sample was labeled representing the plant source (*Sesuvium portulacastrum*: SP) followed by the abbreviation of collection site (P: Parangipettai coastal soil; TV: TEWLIS area Vannapatti series and TT: TEWLIS area Thulukanur series), E in case of Endophytic, and the isolate number.

### 2.3. Halotolerant assay

Initially, all bacterial isolates were screened for halotolerance using NA media supplemented with 2 and 3% NaCl (14.7 and 21.6 dS m^−1^, respectively). The cultures that were found to grow in 3% were selected for further evaluation with growth curve experiment. The 48 h old cultures of the isolates from the broth were transferred with an equal quantity of inoculum (Optical density of 660 nm = 0.10) to 100 mL NA broth supplemented with 3, 5 and 7% NaCl (21.6 dS m^−1^, 32.3 dS m^−1^ and 41.1 dS m^−1^, respectively). The OD 660 nm values were measured once in 4 h for all the isolates until the stationary phase is achieved. The OD 660 values were plotted against the time to obtain the growth curve under each level of NaCl.

### 2.4. Plant growth promotion assessment

Plant growth promoting traits of bacterial isolates were assessed for ammonia production, inorganic phosphate solubilization, siderophore production, IAA production and ACC deaminase activity. All assays were carried out in triplicates and the activity was assessed.

#### 2.4.1. Ammonia production

The production of ammonia by rhizobacteria was tested in 10 mL of peptone water. After 48 h of incubation at 30°C, Nessler’s reagent (0.5 mL) was added to each tube (Bhavani and Kumari, 2019). The development of brown to yellow color was quantified using spectrophotometer against standard graph (425 nm).

#### 2.4.2. Phosphate solubilization assay

The ability of inorganic phosphate solubilization was conducted by spot inoculation of bacterial isolates on modified Pikovskayas agar plates using tricalcium phosphate as a substrate (Goswami et al., 2014). The formation of transparent halo zones around the bacterial colonies after 7 days of incubation at 28°C was considered an indication of phosphate solubilizing activity. The solubilization index was calculated by Equation 1.

(1) $$\mathrm{Solubilization index}=\frac{\left(\mathrm{Colony diameter}+\mathrm{Halo zone diameter}\right)}{\mathrm{Colony diameter}}$$

#### 2.4.3. Siderophores production assay

The siderophore production was assayed by spot inoculation of selected bacterial isolates on Chrome Azurol S (CAS) blue agar plates as described by Schwyn and Neilands (1987). The cultures were incubated for 7 days at 28°C on CAS blue agar plates. The formation of orange zones around the growing colonies was monitored and bacterial isolates scored as siderophore producers.


(2) $$\begin{array}{l} \mathrm{Siderophore production index}=\\ \frac{\left(\mathrm{Colony diameter}+\mathrm{Orange zone diameter}\right)}{\mathrm{Colony diameter}} \end{array}$$

#### 2.4.4. Indole acetic acid production assay

Bacterial isolates were inoculated into 5 mL Luria-Bertani (LB) broth containing 0.2% L-tryptophan, pH 7.0 and incubated at 28°C with shaking at 125 rpm for 7 days. The cultures were centrifuged at 11,000 rpm for 15 min. One milliliter of the supernatant was mixed with 2 mL of Salkowski reagent and the appearance of a pink color indicated IAA production. OD was read at 530 nm using spectrophotometer. The level of IAA produced was estimated against a standard IAA (Shahzad et al., 2017).

#### 2.4.5. ACC deaminase assay

The isolates were grown in 5 mL of LB broth at 28°C until they reached the stationary phase. To induce ACC deaminase activity, the cells were collected by centrifugation and washed twice with 0.1 M Tris–HCl (pH 7.5). Then the cells were suspended in 2 mL of modified DF minimal medium supplemented with 3 mM final concentration of ACC and incubated at 28°C with shaking for another 36–72 h. ACC deaminase activity was determined by measuring the cleavage of ACC into α-ketobutyrate and ammonia. The induced bacterial cells were harvested by centrifugation at 7,500 rpm for 5 min, washed twice with 0.1 M Tris–HCl (pH 7.5), and resuspended in 200 μL of 0.1 M Tris–HCl (pH 8.5). The cells were labialized by adding 5% toluene (v/v) and then vortexed at the highest speed for 30 s. 50 μL of labialized cell suspension was incubated with 5 μL of 0.3 M ACC in an Eppendorf tube at 28°C for 30 min. The negative control for this assay included 50 μL of labialized cell suspension without ACC, while the blank included 50 μL of 0.1 M Tris–HCl (pH 8.5) with 5 μL of 0.3 M ACC. The samples were then mixed thoroughly with 500 μL of 0.56 N HCl by vortexing and the cell debris was removed by centrifugation at 12,000 rpm for 5 min. A 500 μL aliquot of the supernatant was transferred to a glass test tube and mixed with 400 μL of 0.56 N HCl and 150 μL of DNF solution (0.1 g of 2,4-dinitrophenylhydrazine in 100 mL of 2 N HCl) and the mixture was incubated at 28°C for 30 min. One milliliter of 2 N NaOH was added to the sample before the absorbance at 540 nm was measured. The concentration of α-ketobutyrate in each sample was determined by comparison with a standard curve generated as follows: 500 μL α-ketobutyrate solutions of 0, 0.01, 0.05, 0.1, 0.2, 0.5, 0.75, and 1 mM were mixed, respectively, with 400 μL of 0.56 N HCl and 150 μL DNF solution. One milliliter of 2 N NaOH was added and the absorbance at 540 nm was determined as described above. The values for absorbance versus α-ketobutyrate concentration (mM) were used to construct a standard curve (Glick, 2014; Del Carmen Orozco-Mosqueda et al., 2019).

### 2.5. Identification through 16S rRNA sequencing

The bacterial isolates were confirmed by molecular analysis of the 16S rRNA gene for bacteria. DNA isolation from microbial samples was done using the EXpure Microbial DNA isolation kit developed by Bogar Bio Bee stores Pvt. Ltd. DNA concentrations were measured by Qubit fluorometer 3.0. The PCR amplification was done by adding 5 μL of isolated DNA in 25 μL of PCR reaction solution (1.5 μL of Forward Primer and Reverse Primer, 5 μL of deionized water, and 12 μL of Taq Master Mix). The DNA template is heated to 95°C for 2.5 min and cooled at 55°C for 30 s. Then it is heated to 72°C, the optimal temperature for DNA polymerization. The unincorporated primers and dNTPs from PCR products were removed by using Montage PCR Clean up kit (Millipore). Then the DNA sequencing was performed using an ABI PRISM^®^ Big Dye™ Terminator Cycle Sequencing Kit with AmpliTaq^®^ DNA polymerase (FS enzyme) (Applied Biosystems). Single-pass sequencing was performed using below 16 s rRNA universal primers. The samples were resuspended in distilled water and subjected to electrophoresis in an ABI 3730xl sequencer (Applied Biosystems). The 16 s rRNA sequence was blasted using NCBI blast similarity search tool. The phylogeny analysis with the closely related sequence of blast results was performed by multiple sequence alignment. The MUSCLE 3.7 was used for multiple alignments of sequences (Edgar, 2004). Poorly aligned positions and divergent regions were cured using the program G blocks 0.91b (Talavera and Castresana, 2007). Finally, the program PhyML 3.0 aLRT was used for phylogeny analysis and HKY85 as substitution model. The program Tree Dyn 198.3 was used for tree rendering (Dereeper et al., 2008).

### 2.6. *In vivo* evaluation of the selected isolates on *Vigna mungo* L. under salinity

The salt tolerant isolates were evaluated for their effect on germination and growth attributes of *Vigna mungo* L. under salinity (2% NaCl). An initial standardization experiment was carried out to fix the level of initial tolerance of *Vigna mungo* L. Accordingly, germination and growth of *Vigna mungo* L. under three different salinity was evaluated (0.5, 1, 1.5, 2, 3% NaCl). As per ISTA (International Seed Testing Association), the NaCl concentration at which the germination percentage falls below 65% should be selected to observe the impact of microbial inoculation. Hence 2% NaCl concentration was selected for the *in vivo* evaluation of isolates. The *Vigna mungo* L. seeds were surface sterilized in 0.1% sodium hypochlorite for 3 min and repeatedly washed with distilled water. After this, the 28 h old inoculum of all the isolates was individually used for seed priming. The NA broth without isolate was used as a control. The microbial inoculum with an OD of 1 at 660 nm was used for seed priming. After which the seeds were grown in germination sheets placed in 2% NaCl solution. The germination percentage, root length, shoot length and total dry weight were measured after 20 days. The growth vigor was calculated using Equation 3.

(3) $$\mathrm{Vigor index}=\mathrm{Germination percentage}\;x\;\mathrm{Seedling length}\;\left(\mathrm{mm}\right)$$

### 2.7. Compatibility evaluation among the isolates for developing consortium

The culture compatibility among the bacterial isolates was assessed by cross streaking method using NA medium. The bacterial strain was streaked at one end of the plate followed by streaking the other bacterial strains perpendicular to it and incubated at 30°C for 24–48 h. The inhibition in growth between the cultures was noted and the compatible microbes were selected for further confirmation. The selected compatible strains were streaked in a triangular pattern so that all the streaks overlap each other as a confirmation test. Any two perpendicular steaks that showed inhibition were incompatible and the absence of inhibition proved that the cultures were compatible (Thomloudi et al., 2019).

The elite compatible microbial isolates were mass multiplied as pure cultures in the NA media for further formulation of the microbial consortium. The mass culturing was carried out in 250 mL Erlenmeyer flask, which serves as seed culture for further multiplication. The culture broth was autoclaved in 15 lbs for 20 min and after cooling the microbial isolates were inoculated as loopful cultures in their respective broths prepared. The inoculated flasks were maintained as shake flask cultures by incubating them at room temperature at 120 rpm for 2 days for mass production. The 48 h old cultures were serially diluted and plated to count the population in the broth. To attain a uniform population in the consortium, 5 mL inoculum was centrifuged at 10,000 rpm for 15 min at 10°C and resuspended in 10 mL of deionized water to obtain 4 × 10^9^ CFU (Jain and Srivastava, 2012). The OD 660 nm value was also recorded for the resuspended water used for consortium development. Talc was used as carrier material for the consortium due to its local availability and high Magnesium and Calcium content. Talc is a fine, light-weight powder that is easily soluble in water and has been shown to retain viable bio-inoculant propagules (Tripathi et al., 2015). A 100 g of talc was taken in autoclavable plastic bags and autoclaved. A 10 mL of the resuspended solution of each selected isolate was mixed to obtain the consortium. A total volume of 30 mL of the bacterial isolates was added to the carrier material and left for drying in shade (1 day), after which it was evaluated as a soil inoculant.

### 2.8. Evaluation of microbial consortium application on growth and yield of *Vigna mungo* L.

The potential of microbial consortium dosage on alleviation of salt stress in *Vigna mungo* L. was assessed through a pot culture experiment and the details of treatment are given in Table 1. The treated effluent with EC of 2.5 dS m^−1^ was used for irrigation. The pots were irrigated at an interval of 5–7 days based on the field capacity of the soil (22.5 per cent) which was estimated using pressure plate apparatus (Obi, 1974). After the application of amendments and microbial consortium, five seeds were sown in each 3 kg pot and irrigated with treated effluent. Thinning was carried out after 15 days to maintain a uniform population of 3 plants per pot. The morphological, physiological and biochemical parameters were recorded at three growth stages *viz*., vegetative (25th day), flowering (45th day) and harvest stage. The height of the plant from the ground level to the tip of the main stem was measured and expressed in centimeters. The physiological and biochemical parameters including chlorophyll content, leaf free proline content and catalase were carried out using standard procedures.

**Table 1:** Details of treatment for assessing the salinity stress alleviation by microbial consortium.

Factor 1:	Microbial consortium
I_1_ -	Control
I_2_ -	Microbial consortium I (*M. indicus* + *N. niacini* + *S. marcescens*)
I_3_ -	Microbial consortium II (*B. velezensis* + *K. radicincitans* + *K. rhizophila*)
Factor 2:	Dosage
D_1_ -	2 kg ha^−1^ as soil application
D_2_ -	4 kg ha^−1^ as soil application

#### 2.8.1. Morphological and physiological characterization of plant samples

The morphological and physiological characteristics of the plant were measured during the flowering stage of the crop (45 days after sowing). The Chlorophyll Content Meter (CCM-200+, United States) was used to assess chlorophyll content in the leaves. These measurements were taken at three different points of young fully expanded leaves of each treatment during bright sunshine (Dhevagi et al., 2022). Similarly, photosynthetic rate and stomatal conductance were measured using a portable photosynthesis system (ADC BioScientific LCpro-SD System, United Kingdom) (Dhevagi et al., 2022).

#### 2.8.2. Osmoregulatory metabolite and antioxidant enzymes analysis

The biochemical parameters like proline, catalase, and superoxide dismutase (SOD) were assessed during the flowering stage of the crop (45 days after sowing). For estimation of proline, 0.5 g of fresh leaf was homogenized in chilled 10 mL of 3% sulfosalicylic acid in precooled pestle and mortar. The homogenate was centrifuged at 12,000 rpm for 5 min at 4°C and 2 mL supernatant was transferred to test tube. Then 2 mL acid ninhydrin and 2 mL of glacial acetic acid were added and incubated in water bath (100°C) for 1 h. Then the test tubes were placed in ice bath to stop the reaction. Four mL of toluene was added and the tube was vortexed for 1 min. The extract was transferred to a cuvette to record the absorbance at 520 nm and plotted against standard graph of proline to express as μmol g^−1^ fresh weight (Bates et al., 1973). The catalase activity was estimated by following the methodology of Gopalachari (1963). Fresh plant tissue of 5 g was homogenized with ice-cold phosphate buffer (PB) 5 ml and centrifuged at 12,000 rpm for 10 min at 4°C to obtain the supernatant. One mL of supernatant was mixed with 1 mL of PB and 1 mL of 30 mM hydrogen peroxide and the absorbance of the supernatant was read at 240 nm. The decrease in absorbance was recorded every 30 s for 5 min. The absorbance was plotted against the standard graph of hydrogen peroxide to assess the catalase activity as the rate of oxidation of hydrogen peroxide per minute per gram. For SOD, one gram of fresh leaf was macerated in 10 ml of chilled 50 mM phosphate buffer in a prechilled mortar and pestle. The mixture was centrifuged at 12,000 rpm for 10 min at 4°C to collect the supernatant. A reaction mixture of 1.3 mL of sodium carbonate buffer, 500 μL of Nitroblue tetrazolium (NBT) and 100 μL EDTA was prepared. After which 100 μL of hydroxylamine hydrochloride was added and incubated for 2 min at room temperature to initiate the reaction. 100 μL of supernatant was added and the absorbance at 560 nm was recorded every 30 s for 1–2 min. The absorbance was plotted against the standard graph of NBT at 560 nm to obtain the superoxide dismutase activity per gram of sample (Dhindsa et al., 1981).

#### 2.8.3. Assessing the quantity and quality of yield

The mature pods in *Vigna mungo* L. were harvested after the 65th day and expressed in g pot^−1^. The harvest index used as a measure of reproductive efficiency was worked out by using Equation 4. The protein content in the seeds was assessed through Folin phenol reagent method (Lowry et al., 1951). About 250 mg of *Vigna mungo* L. pod sample was taken and macerated with 10 mL of phosphate buffer solution. Centrifuged the contents at 3,000 rpm for 10 min and the supernatant was collected and made up the volume to 25 mL with distilled water. To 1 mL of the supernatant taken in a test tube, 5 mL of Lowry reagent and 0.5 mL of Folin reagent were added and incubated for 30 min. The color development was measured at 660 nm in a UV–vis spectrophotometer and bovine serum albumin was used as standard.


(4) $$\mathrm{Harvest index}=\frac{\mathrm{Economic yield}}{\mathrm{Biological yield}}$$

### 2.9. Statistical analysis

The data on various characteristics studied during the investigation were statistically analyzed by the method given by Gomez and Gomez (1984) using SPSS Version 16.0. The critical difference was worked out at 5 per cent (0.05) probability levels.

## 3. Results and discussion

### 3.1. Isolation of halotolerant bacteria from rhizosphere and root endosphere of *Sesuvium portulacastrum*

A total of eight morphologically different bacterial strains were isolated from rhizospheric soil samples of *Sesuvium portulacastrum* collected from coastal areas of Parangipettai. Bacterial isolates were labeled as SPP 1 to SPP 8. The rhizosphere soil samples from paper and pulp mill effluent irrigated soil (TEWLIS area) were collected and 16 strains with varying morphology were isolated. Out of 16 isolates, 9 were from Thulukanur soil series (SPTT 1 – SPTT 9) and 7 were from Vannapatti soil series (SPTV 1 – SPTV 7). Similarly, eight endophytic bacterial isolates were obtained from roots of profusely grown *S. portulacastrum* cultivated in two soil series of paper and pulp mill effluent irrigated areas. Two strains from Thulukanur soil series were named SPTTE 1 and SPTTE 2. Similarly, five strains from Vannapatti soil series were isolated and named SPTVE 1 to SPTVE 5. The isolates were screened for salt tolerance in NA media supplemented with 3% NaCl, since identifying halotolerant PGPR with high tolerance potential is essential. The inoculated plates were observed for colony growth after 48 h of incubation. Among 31 bacterial isolates, profuse growth was observed in 9 isolates (SPP 2, SPP 5, SPP 6, SPTT 3, SPTT 7, SPPTT 8, SPTV 3, SPTVE 3, and SPTVE 4), whereas other strains failed to grow in NA media supplemented with 3% NaCl. Among the isolates, two (SPTVE 3 and SPTVE 4) were endophytes.

### 3.2. Morphological and biochemical characteristics of bacterial isolates

The selected 9 strains were characterized for cell morphology, Gram behavior, pigment production and motility (Supplementary Table S2). Out of 9 isolates, 6 tested positive in Gram test and the remaining were Gram negative. The isolate SPTT 8 was coccoid in shape and all others were rod shaped. The isolates SPP 2 and SPTT 8 exhibited orange and light green colonies, respectively, whereas no distinguishable colors were noticed in the other isolates. The orange or red pigmentation, due to carotenoids might help the bacteria against damaging UV radiation (Khaneja et al., 2010). Strains SPTT 3, SPTT 7, SPTVE 3, and SPTVE 4 were motile which has diffused zone of growth extended out from the line of inoculation. Both flagellar motility and citrate utilization are thought to be important in bacterial root colonization and maintenance (Weisskopf et al., 2011). Biochemical characters such as casein, starch hydrolysis, citrate, amylase, protease, urease, catalase and oxidase activities, indole production, nitrate reduction and Methyl Red test – Voges Proskauer (MR-VP) are given in Supplementary Table S2. Among these traits, protease, catalase and amylase were positive for all the isolates. MR-VP test was positive for all isolates except SPP 2, SPP 5 and SPTVE 3. Isolates SPP 6, SPTT 8 and SPTVE 3 tested positive for urease activity. The strains SPP 5, SPTT 3, SPTT 7 and SPTVE 3 had the ability to reduce nitrate to nitrite. All the isolates tested positive for catalase test which indicates all are aerobic bacteria and would neutralize hydrogen peroxide by producing catalase enzyme (Facklam and Elliott, 1995). Protease test was positive for all the isolates which indicates their ability to breakdown protein into amino acids.

### 3.3. Identification of PGPR isolates by 16S rRNA sequence analysis

**Figure 2:**
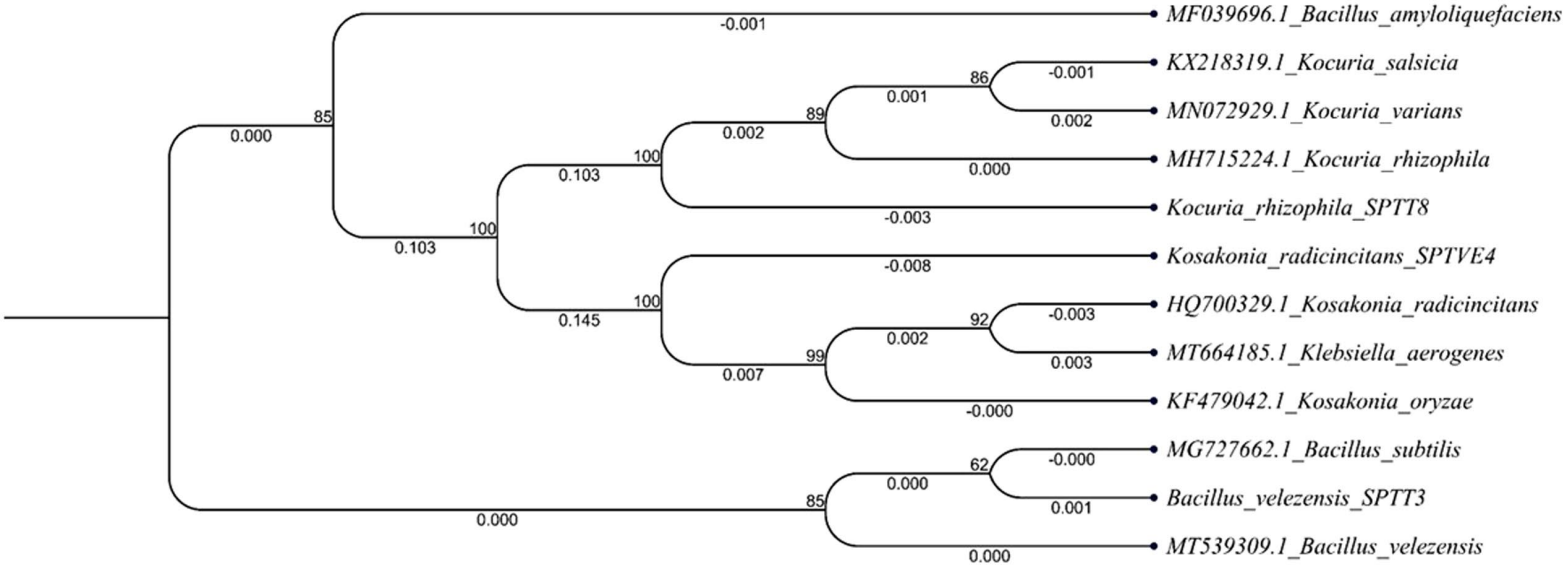
Phylogenetic tree of halotolerant isolates used for developing microbial consortium – II (*Bacillus velezensis*; *Kocuria rhizophila* and *Kosakonia radicincitans*). The 16S rRNA gene sequences of closely related species were retrieved from NCBI GenBank databases. The Neighbor joining phylogenetic tree was inferred using MEGA-7 software; evolutionary distance was computed using Maximum Composite Likelihood method at bootstrap value of 1,000.

The nine selected bacterial isolates were identified by 16S rRNA sequencing and blasting in the NCBI database. The isolate SPP 2 has a similarity (99.9%) with that of *Metabacillus indicus* and SPP 5 was 99.7% similar to *Neobacillus niacini*. The SPP 6 was 100% similar to *Staphylococcus warneri* and SPTT 3 was 99.8% similar to *Bacillus velezensis*. Similarly, SPTT 7, SPTT 8 and SPTV 3 were similar to *Bacillus circulans, Kocuria rhizophila* and *Bacillus oleronius*, respectively. The isolate SPTVE 3 had 99.9% similarity with *Serratia marcescens* and SPTVE 4 was 99.8% similar to *Kosakonia radicincitanas*. The phylogenetic tree of isolates used in microbial consortium II was obtained by the Neighbor Joining method with a boot strap value of 1,000 and depicted in Figure 2. The derived nucleotide sequence was submitted to NCBI database and the GenBank accession numbers are given in Table 2.

**Table 2:** 16S rRNA identification of the halotolerant plant growth promoting bacterial isolates.

Strain	Best match	Accession number	Match index
SPP 2	*Metabacillus indicus*	OP836561.1	99.6%
SPP 5	*Neobacillus niacini*	OP836562.1	99.7%
SPP 6	*Staphylococcus warneri*	OP836563.1	100%
SPTT 3	*Bacillus velezensis*	OP836557.1	99.8%
SPTT 7	*Bacillus circulans*	OP836560.1	100%
SPTT 8	*Kocuria rhizophila*	OP836556.1	100%
SPTV 3	*Bacillus acidicola*	OP836559.1	99.2%
SPTVE 3	*Serratia marcescens*	OP836558.1	99.9%
SPTVE 4	*Kosakonia radicincitans*	OP836555.1	99.8%

### 3.4. Assay for *in vitro* plant growth promotion activity

#### 3.4.1. Indole-3-acetic acid (IAA) production

The bacterial isolates were screened for their ability to produce auxin (IAA). IAA is a phytohormone that functions as a natural auxin in plants (Carmen and Roberto, 2011). By using L-tryptophan found in root exudates, several bacteria can produce IAA as a secondary metabolite. IAA-producing bacteria help plants maintain their growth under salt conditions by increasing root length. Except for *S. warneri*, all other isolates produced red color after the addition of Salkowski’s reagent in the culture supernatant since IAA production was found very common in PGPR, as shown by similar studies (Galkovskyi et al., 2012; Zahid et al., 2015; Islam et al., 2016). However, the intensity of red color developed was very low without tryptophan in the isolates *N. niacini* and *B. olerenius*. With the addition of tryptophan in the broth, IAA production was enhanced. The isolate SPTVE 3 had the highest IAA production of 23.60 μg mL^−1^ followed by SPP 2 (22.80 μg mL^−1^). The lowest IAA production was recorded in isolate *B. olerenius* (9.41 μg mL^−1^) in tryptophan supplemented medium (Table 3). Devi et al. (2016) reported that *Serratia marcescens* AL2-16 isolated from *Achyranthes aspera* L. produced 83.2 μg mL^−1^ of IAA in the presence of 1% L-tryptophan after 24 h incubation. Similarly, *S. marcescens* isolated in this study also produced the highest IAA (23.60 μg mL^−1^ in 0.2% L-tryptophan) among other isolates.

**Table 3:** Plant growth promotion capabilities of the bacterial isolates from the rhizosphere and endosphere of *S. portulacastrum*.

Strains	Consortium	Phosphate solubilization index	Siderophore production index	IAA production (μg mL^−1^)		Ammonia production (μg mL^−1^)	ACC deaminase activity (μM of α-ketobutyrate released mg^−1^ protein h^−1^)
				2 mg tryptophan g^−1^ broth	No tryptophan		
*M. indicus*	MC-I	2.44	–	22.81	4.20	3.86	0.603
*N. niacini*		–	2.88	10.40	1.70	–	–
*S. marcescens*		2.25	2.13	20.52	3.70	2.83	0.751
*B. velezensis*	MC-II	2.29	1.89	14.43	2.15	4.28	1.180
*K. radicincitans*		2.63	2.50	23.60	5.35	5.19	0.395
*K. rhizophila*		2.50	2.22	11.23	2.25	3.40	0.983
*S. warneri*	Non-compatible	2.00	1.70	–	–	–	–
*B. acidicola*		2.22	1.19	9.41	1.60	–	–
*B. circulans*		–	2.29	12.69	2.60	2.60	0.324

#### 3.4.2. Phosphate solubilization

The presence of P-solubilizers in soils may be seen as positive signs of using microorganisms as biofertilizers to enhance crop yield and promote sustainable agriculture development. The production of microbial metabolites such as organic acids, which lower the pH of the media, is primarily responsible for phosphate solubilization (Shahid et al., 2012). Phosphate solubilization by the isolates was visualized by the development of clear zones (Halo-zone) around the bacterial colonies after 3 days of incubation in Pikovskayas medium (Supplementary Figure S1). Out of 9, seven isolates were phosphate solubilizers indicated by the clear zones. The highest index was observed in the isolate *K. radicincitans* (2.63), followed by *K. rhizophila* at 2.50 (Table 3). The isolates *N. niacini* and *B. circulans* tested negative to solubilize phosphate. Shahid et al. (2015) stated that the most commonly exploited phosphate solubilizers are bacteria belonging to *Pseudomonas* sp., *Bacillus* sp., *Rhizobium* sp. and *Enterobacter* sp. Though *N. niacini* and *B. circulans* isolated in this study, belong to the bacillus genera they lack the ability to solubilize phosphate. *K. radicincitans* of Enterobacteriaceae family had the highest phosphate solubilization index (2.63) than the other isolates. Afridi et al. (2019) observed 16–17 mm halo zone around the colony of *Kocuria rhizophila*, whereas in the present study, 12 mm halo zone was formed in 48 h. Phosphate solubilizing bacteria have the ability to enhance phosphorus availability in the soil thereby promoting crop growth. In some cases, phosphate solubilization bacteria can promote plant growth both indirectly and directly by reducing phytopathogen growth and synthesizing phytohormones (Bhattacharjee, 2012).

#### 3.4.3. Siderophore production

Siderophores are chemical compounds with iron-specific ligands that have low molecular weight (<10,000 Da). Various microorganisms synthesize it as an iron scavenging agent to maintain minimal iron stress. They are infinitesimal iron chelating compounds with a strong affinity for transporting ions across cell membranes and disease control (Pandey et al., 2017). Several studies have shown that the rhizosphere micro flora produces siderophores, which improve plant iron uptake (Kotasthane et al., 2017; Priyanka et al., 2020). Siderophore activity was absent in strain *M. indicus*, while all other isolates had production index in the range between 2.88 and 1.19 (Table 3). *Bacillus velezensis* NRRL B-41580 and *Bacillus siamensis* KCTC 13613 isolated from the rhizosphere of rice, respectively produced 69 and 55 per cent siderophore, according to Masum et al. (2018). Navarro-Torre et al. (2016) observed in their study that *S. warneri* isolated from the halophyte, *Arthrocnemum macrostachyum* had shown no siderophore activity. Contrastingly, in our study, the isolate *S. warneri* had a siderophore production index of 1.70. *Pantoea dispersa* isolated from mung bean rhizosphere had a 6–9 mm halo zone on Pikovskaya’s agar medium indicating their ability to produce siderophore (Panwar et al., 2016; Supplementary Figure S2). The environmental conditions influence the production of bacterial siderophores and their efficacy in transferring iron (Singh et al., 2022). The siderophore increases the rate of phytoextraction of metals from rhizosphere. The production and transport of siderophore vary between Gram positive and Gram negative bacteria, since the outer membrane transporters are broadly absent in Gram positive bacteria. These transporters play a vital role in the transport of Fe-siderophore. RS-I (Reduction strategy) and CS-II (Chelation strategy) are two types of mechanism for Fe transport into the plant system. While, RS-I strategy is predominantly found in iron deficit soils, CS-II strategy is found in alkaline soil where bacteria are important agents for improving iron availability. This strategy (CS-II) is based on biosynthesis of siderophore that chelates Fe and transport it through TOM1 (Translocase of Outer Membrane) to the root (Dai et al., 2018).

#### 3.4.4. Ammonia production

Ammonia production plays an important role in increasing plant growth by accumulating nitrogen, as well as assisting in root, shoot and biomass development (Dutta and Thakur, 2017). It also plays a vital role in remediation of polluted environment (Raklami et al., 2021), carbon sequestration (John and Lakshmanan, 2018) and various ecosystem services (Razzaghi Komaresofla et al., 2019). The highest value of 5.19 μg mL^−1^ was recorded by isolate *K. radicincitans*, followed by *B. velezensis* (4.28 μg mL^−1^). Ammonia production was absent in the isolates *N. niacini*, *S. warneri*, and *B. olernius*. Ammonia production in *K. rhizophila* isolated from *Oxalis corniculata* was reported to be 36 μg mL^−1^ by Afridi et al. (2019), whereas in this study it was only 2.60 μg mL^−1^ which is the lowest ammonia production among other isolates. The isolate *K. radicincitans* produced the highest ammonia (5.79 μg mL^−1^), followed by *B. velezensis* (4.8 μg mL^−1^) (Table 3). The accumulation of ammonia in soil disrupts the microbial community’s homeostasis and prevents the germination of many fungal spores thereby adding many beneficial attributes (Gupta and Pandey, 2019).

#### 3.4.5. ACC deaminase activity

Ethylene is produced in plant systems under stress conditions which have detrimental effects on crop growth. ACC deaminase produced by the bacterial isolates degrades ACC, a precursor of ethylene into α-ketobutyrate. Hence, they help plants to assuage the ethylene-induced effect on growth and development, particularly under salt stress (Arora, 2020). The highest activity was recorded in the isolate *B. velezensis* (1.180 μM mg^−1^ protein h^−1^) and followed the order of *K. rhizophila* > *S. marcescens* > *M. indicus* > *K. radicincitans* > *B. circulans* (Table 3). The ACC deaminase activity was not recorded in the isolates *N. niacini*, *S. warneri* and *B. oleronius*. The breakdown products of ACC, notably α-ketobutyrate and ammonia, provide nitrogen and energy to the microorganisms that are involved in the degradation. The ACC deaminase activity of 853–2,107 nmol ketobutyrate mg protein^−1^ h^−1^ was observed in PGPR strains belonging to *Pseudomonas* sp. and *Bacillus* sp. isolated from halophyte *Atriplex* sp. (Leontidou et al., 2020). They also suggested that plants thriving in extreme conditions like saline or drought stress environment might be the primary selectors of bacterial ACC deaminase activity.

### 3.5. Salinity tolerance of selected bacterial isolates under different salinity levels (0, 5 and 7% NaCl)

**Figure 3:**
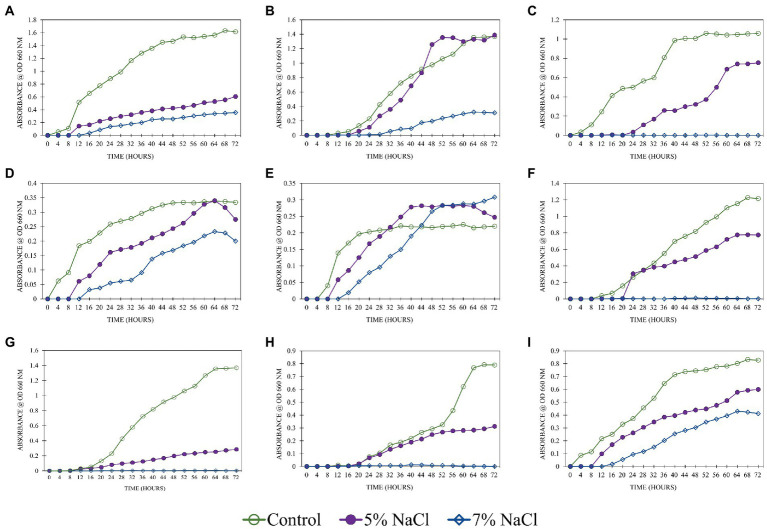
Salinity tolerance of the bacterial isolates under different levels of NaCl (0% -control, 5%, 7%) A: SPP 2 - *M. indicus*, B: SPP 5 - *N. niacini*, C: SPP 6 - *S. warneri*, D: SPTT 3 - *B. velezensis*, E: SPTT 7 - *B. circulans*, F: SPTT 8 - *K. rhizophila*, G: SPTV 3 - *B. oleronius*, H: SPTVE 3 - *S. marcescens* and I: SPTVE 4 - *K. radicincitans*.

The salinity levels had a profound influence on the growth of isolates tested for tolerance (Figure 3). All the isolates had shown profound growth in control. The highest growth rate was observed in isolate SPTT 7. Including control, this isolate had grown well at both concentrations of NaCl (5 and 7%). In 7% NaCl, the stationary phase was attained at 52 h after inoculating. The isolates SPP 5 and SPTT 8 were unable to grow at 7% NaCl concentration and the growth rate slackened at 5% NaCl. *M. indicus* and *B. oleronius* had very slow growth rate at both the NaCl concentrations (5 and 7%) than the control. But the growth rate declined after 64 h in 5 and 7% NaCl. At 5% NaCl, *S. marcescens* had a slow and steady growth rate, while at 7% no growth was observed. In control, the lowest OD 660 nm value of 0.35 was recorded in the isolate *B. velezensis* at 60 h. The growth rate declined after 64 h at both NaCl concentrations (5 and 7%). The isolate *S. warneri* attained stationary phase after 40 and 60 h in control and 5% NaCl, respectively. High concentration of NaCl (7%) restricted the growth of the isolate. Isolates *S. warneri, K. rhizophila* and *S. marcescens* failed to grow at 7% NaCl, while they had a steady growth rate at 5%. Singh and Jha (2016b) also reported that *S. marcescens* grew well up to 6% NaCl. Similarly, *K. rhizophila* isolates had been reported to grow substantially in 5% salt medium, however, when salt concentration increased, the isolates showed a decreasing trend (Shi et al., 2021). *B. circulans* and *B. oleronius* had a shorter lag phase of 8 h than the other isolates, which indicated the potential of the microbe to grow under saline conditions (Finkel and Kolter, 1999). All the *Bacillus* genera (*B. oleronius*, *B. circulans*, *N. niacini*, and *M. indicus*) had long stationary phase that could be due to the synthesis of protective factors and adaptation of current environmental conditions at higher NaCl concentrations. In a study conducted by Duc et al. (2006), *B. indicus* tolerated higher levels of NaCl (8%), similarly *Metabacillus indicus* isolated in this study also tolerated 7% NaCl. Identifying halotolerant PGPR that could tolerate high levels of salinity is imperative for improving crop productivity under severe stress.

### 3.6. The modulation of metabolites and enzymes in plants under saline stress

High salt concentration in the rhizospheric zone leads to osmotic stress and disrupts cell ion homeostasis due to the accumulation of Na^+^ and Cl^−^ ions (Paranychianakis and Chartzoulakis, 2005). This interferes with the uptake of essential nutrients and water thereby inhibiting plant growth. It affects shoot growth as well as xylem and root architecture. The root architecture in sense, the biomass, root network length and root length density are significantly altered, amplifying the impacts of salinity in the plants (Machado and Serralheiro, 2017). Plants cope with this by secretion of metabolites like proline and upregulation of potassium uptake that regulate homeostasis. The proline is synthesized from L-glutamate which requires ATP and NADH. Additionally, oxidative stress due to generation of ROS alters various biochemical pathways by inhibiting the binding of enzymes with substrate (Singh et al., 2022). Both osmotic stress and ionic stress result in oxidative damage to membrane components and cell organelles especially, chloroplasts. Owing to this the chlorophyll content in the plants under saline stress is reduced (Fatma et al., 2021). This inhibits photosynthetic activity and protein production, followed by inactivation of critical enzymes in the ATP synthesis (Singh et al., 2022). Nucleic acids, the structural component of proteins and DNA are impaired, leading to seizure of replication or transcription process (Hasanuzzaman et al., 2021). The chlorophyll degradation due to salinity is very high with the prolongation of stress (Hossain et al., 2017). In general, about 80% of the growth reduction at high salinity could be attributed to ethylene build up, resulting in reduction of leaf area, loss of chlorophyll content and decline in photosynthesis. The remaining 20% could be likely explained by a decrease in stomatal conductance and nutrient non-availability (Isayenkov and Maathuis, 2019). Ethylene is a plant hormone that regulates root hair, root growth, fruit ripening, leaf abscission, seed germination and ROS in the plant system (Fatma et al., 2021). Consequently, these changes in the plant’s metabolism could reduce the growth and yield of crops. Hence, the alleviation of above impacts with the aid of microbiome could help the crop to tolerate the salinity and increase yield even under high saline environments.

### 3.7. Effect of isolated saline tolerant PGP bacterial strains on germination of *Vigna mungo* L. seeds under *in vitro* conditions (with 2% NaCl)

The results of *in vitro* experiment also revealed that when inoculated with the microbial isolates, the test plant’s growth improved significantly even under salt stress (Table 4). Significant difference in germination percentage, root length, shoot length and vigor index of *Vigna mungo* L. seeds was noted. Each isolate showed varying degrees of enhancement in crop growth attributes. Among the treatments, the highest germination percentage (88.5%) of *Vigna mungo* L. seeds was reported in *S. marcescens* inoculated treatment (T_10_) and the lowest was in (T_2_) uninoculated control (2% NaCl) with 65.3%. Treatments inoculated with *N. niacini* (T_4_) and *B. velezensis* (T_6_) recorded 83.4 and 85.2% germination, respectively. Though germination percentage was high when inoculated with *S. marcescens* (T_10_), root length was observed to be high (6.8 cm) when inoculated with *B. velezensis* (T_6_). The highest vigor index of 1819 was recorded with *B. velezensis* (T_6_), followed by *N. niacini* (T_4_) inoculation (1785). The uninoculated control (2% NaCl) had the lowest vigor index of 780 followed by *S. warneri* (T_5_) inoculation (792). In an experiment conducted by Singh and Jha (2016b), *S. marcescens* (T_10_) inoculation improved wheat plant growth under salinity stress (150–200 mM) and effectively reduced the suppression of plant growth due to salt stresses. Under salt stress (2% NaCl), the lengths of shoots and roots in uninoculated plants were severely reduced, whereas their lengths increased significantly in the presence of PGPR. The availability of higher auxin concentrations as IAA by these inoculants is likely to be the cause of plants’ increased root length (Rajkumar et al., 2005; Chakraborty et al., 2006). Shukla et al. (2012) also revealed that inoculation of halotolerant PGPR strains isolated from *Salicornia brachiata* improved the groundnut crop growth in hydroponics experiment with 100 mM NaCl than the control. The shoot length (14.6 cm) was observed to be higher in *S. marcescens* and *B. velezensis* inoculated plants, than in the uninoculated control (8.2 cm). Similarly, tomato seeds coated with *B. velezensis* strain had higher plant height and stem diameter at 100 mmol L^−1^ NaCl than the control (Medeiros and Bettiol, 2021). In this study, *K. radicincitans* inoculated treatment recorded high shoot and root growth of 62.2 and 79%, respectively, than the uninoculated control. Similarly, Berger et al. (2013) also reported 80 and 50% increase in root and shoot growth of 5 weeks old tomato plants, emphasizing *K. radicincitans* significance as a strong PGPR for variety of crops.

**Table 4:** *In vivo* evaluation of isolates for salinity stress alleviation in *Vigna mungo* L. under 2% NaCl.

Treatments	Germination percentage (%)	Shoot length (cm)	Root length (cm)	Vigor index
T_1_ – Absolute control	98.3 ^a*^	14.80 ^a*^	7.90 ^a*^	2,270 ^a*^
T_2_ – (2% NaCl)	65.3 ^j*^	8.20 ^h*^	3.80 ^f*^	780 ^h*^
T_3_ – (*M. indicus* + 2% NaCl)	70.1 ^g*^	12.60 ^e*^	7.40 ^a*^	1,225 ^e*^
T_4_ – (*N. niacini* + 2% NaCl)	83.4 ^d*^	14.10 ^b*^	4.90 ^e*^	1785 ^c*^
T_5_ – (*S. warneri* + 2% NaCl)	66.0 ^i*^	8.50 ^g*^	3.50 ^g*^	792 ^h*^
T_6_ – (*B. velezensis* + 2% NaCl)	85.2 ^c*^	14.60 ^a*^	6.80 ^b*^	1819 ^b*^
T_7_ – (*B. circulans* + 2% NaCl)	78.0 ^e*^	13.80 ^c*^	3.20 ^h*^	1,537 ^d*^
T_8_ – (*K. rhizophila* + 2% NaCl)	71.6 ^f*^	11.08 ^f*^	5.10 ^d*^	1,158 ^f*^
T_9_ – (*B. acidicola* + 2% NaCl)	68.0 ^h*^	8.90 ^g*^	5.90 ^c*^	823 ^g*^
T_10_ – (*S. marcescens* + 2% NaCl)	88.5 ^b*^	14.60 ^a*^	5.30 ^d*^	1751 ^c*^
T_11_ – (*K. radicincitans* + 2% NaCl)	75.1 ^e*^	13.30 ^d*^	6.80 ^b*^	1,508 ^d*^
Mean	77.2	12.23	5.51	1,404

Values that do not share similar letters denote statistical significance; * denote significance at *p* < 0.05.

### 3.8. Compatibility and antagonistic activity among the bacterial strains

**Figure 4:**
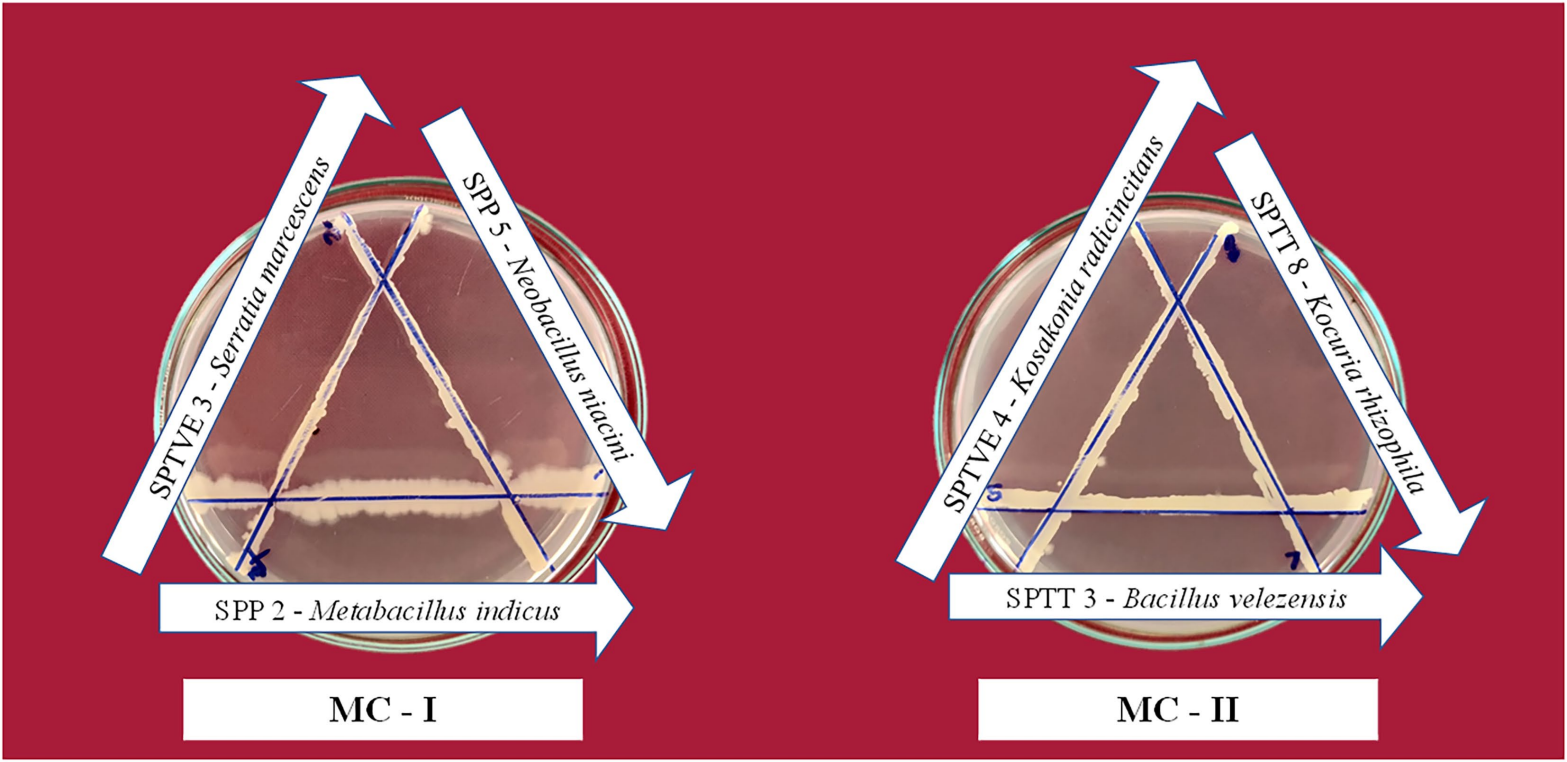
Compatibility assay between the bacterial isolates in the halotolerant microbial consortia. The lack of inhibition zone between two streaks indicate compatibility.

The bacterial isolate in the microbial consortium must be able to proliferate in the presence of each other without hindering the growth and development of other microorganisms. The phytohormone develops a signaling network which mutually regulates several metabolic systems between the microorganisms (Patel et al., 2016). When isolates in a consortium have antagonistic relationships with each other, it makes the consortium unstable, and the intended function is not attained (Sarkar and Chourasia, 2017). Compatibility analysis of different PGPR cultures with each other in line streak assay revealed the isolates antagonistic activity against each other. The absence of an inhibition zone indicated that the isolates were compatible with each other. A zone of inhibition was observed when isolates *B. circulans* and *B. oleronius* were streaked indicating their high antagonistic activity. Isolates *M. indicus*, *N. niacini*, and *S. marcescens* were compatible with each other, since there was no inhibition zone observed between these three isolates. Similarly, *B. velezensis*, *K. rhizophila*, *B. oleronius*, and *K. radicincitans* had compatibility with each other. In secondary confirmation study, *B. oleronius* had shown inhibition zone with *B. velezensis* and *K. radicincitans*. In this study, *B. circulans* had antagonistic effect with all the other isolates. Abada et al. (2014) noted the presence of Permetin A, an antibiotic chemical in *B. circulans* culture filtrate that had activity against Gram negative bacteria. Hence, this could be the reason for incompatibility with other isolates. *M. indicus*, *N. niacin* and *S. marcescens* had synergism between them and were combined as Microbial consortium I. Similarly, Microbial consortium II was formed with *B. velezensis*, *K. rhizophila*, and *K. radicincitans*. These isolates were tested again for their compatibility by streaking them in triangular shape and no inhibition zone was observed between the isolates in both the consortia (Figure 4).

### 3.9. Effect of inoculation of microbial consortium on *Vigna mungo* L. under saline conditions

#### 3.9.1. Inoculation of halotolerant microbial consortium on osmolytes and antioxidant enzymes in *Vigna mungo* L. leaves

The osmolyte, proline and stress enzymes such as catalase and SOD were lower in the microbial consortia inoculated treatments than the control. The lowest proline content of 10.45 μmol g^−1^ FW (I_3_D_1_) was recorded in the treatments inoculated with microbial consortium II (*B. velezensis*, *K. radicincitans* and *K. rhizophila*) at 2 kg ha^−1^ (Table 5). Concurrently, the control I_1_D_1_ followed by I_1_D_2_ had the highest proline contents with 13.86 and 12.87 μmol g^−1^ FW, respectively. The highest proline accumulation was observed in the uninoculated *Vigna mungo* L. seedlings, which was negated in treatments inoculated with the microbial consortium. The mean values ranged from 10.55 (I_3_) to 13.36 μmol g^−1^ FW (I_1_) which indicates a significant reduction in stress in crops inoculated with microbial consortium. Microbial consortia and dosage level were found to have a significant impact on the proline content of *Vigna mungo* L. Especially, the inoculation of MC-II at 2 kg ha^−1^ (24.6%) had the highest reduction in proline content than the MC-II at 4 kg ha^−1^ (17.2%). Plants generally accumulate osmoprotectants such as proline in response to salt stress, which aids in osmotic adjustment and prevents cellular oxidative damage (Shukla et al., 2012; Ilangumaran and Smith, 2017). The disruption in homeostasis under saline conditions could be caused by accumulation of sodium ions in large quantities. The siderophore producing potential of the MC-II is slightly higher than MC-I, which could have sequestered the sodium ions in the root surfaces similar to metals. In addition to that *B. velezensis* in MC-II was noted with high tendency to form mats of growth in the laboratory assays. This could have adsorbed the sodium ions there by reducing their availability around the root zone.

**Table 5:** Effect of halotolerant microbial consortia dosage on stress alleviation in *Vigna mungo* L. under salinity.

Treatments	Proline (μmol g^−1^ FW)			Catalase (mM H_2_O_2_ oxidized min^−1^ g^−1^)			Superoxide dismutase (U g^−1^ FW)		
	D_1_	D_2_	Mean	D_1_	D_2_	Mean	D_1_	D_2_	Mean
I_1_ (Control)	13.86 ^d*^	12.87 ^d*^	13.36	0.28 ^c*^	0.27 ^c*^	0.28	3.24 ^b*^	3.25 ^b*^	3.25
I_2_ (MC-I)	12.48 ^c*^	12.16 ^b*^	12.32	0.26 ^b*^	0.26 ^b*^	0.26	3.22 ^b*^	3.19 ^a*^	3.21
I_3_ (MC-II)	10.45 ^a*^	10.65 ^a*^	10.55	0.25 ^a*^	0.25 ^a*^	0.25	3.20 ^a*^	3.17 ^a*^	3.19
Mean	12.26	11.89	12.08	0.26	0.26	0.26	3.22	3.20	3.21

I_1_ – Control; I_2_ – Microbial consortium I; I_3_ – Microbial consortium II and D1−2 kg of bioformulation per ha; D2−4 kg bioformulation per ha (Values that do not share similar letters denote statistical significance; * denote significance at *p* < 0.05).

The control (I_1_D_1_) had higher catalase activity with 0.28 mM H_2_O_2_ oxidized min^−1^ g^−1^ followed by I_1_D_2_ at 0.27 mM H_2_O_2_ oxidized min^−1^ g^−1^, compared to that of the other treatments (Table 5). This increase in the antioxidant enzymes could be due to the oxidative damage caused by ROS (Kang et al., 2014). The lower catalase activity of 0.25 mM H_2_O_2_ oxidized min^−1^ g^−1^ was recorded in I_3_D_1_ and I_3_D_2_ (inoculated with MC-II). There was no statistically significant difference in catalase activity due to the dosage of the microbial consortium. Remarkably, there is significant difference in the accumulation of SOD between inoculated and uninoculated treatments. The lowest SOD content of 3.17 U g^−1^ FW was recorded in crops inoculated with MC-II (I_3_D_2_). The control I_1_D_2_ followed by I_1_D_1_ had higher SOD content with 3.25 and 3.24 U g^−1^ FW, respectively. Similar investigation by Chanratana et al. (2019) on salt stress alleviation in tomato using *Methylobacterium oryzae* recorded higher catalase and SOD activity in untreated plants than the inoculated. These antioxidants are either up-regulated or down-regulated to reduce abiotic stress depending on the plant and bacteria species. ROS (O_2_^−^, ^1^O_2_, OH^−^ and H_2_O_2_) production was reported to be generated in plants under different types of environmental stresses, such as high or low temperature, salinity, drought, nutritional inadequacy, and pathogen attack (Singh et al., 2019). The ethylene signaling modulates salinity responses largely *via* regulation of ROS-generation in plant system (Zhang et al., 2016). The ACC deaminase activity by the microbial consortia reduces the percussor of ethylene leading to less ROS production. Owing to this the antioxidant enzymes, catalase and SOD activity have been reduced in crops inoculated with microbial consortia. Due to high ACC deaminase activity potential of MC-II (Table 2), the reduction of antioxidants in crops inoculated with MC-II is significantly lower than MC-I inoculated.

#### 3.9.2. Effect on growth attributes of *Vigna mungo* L. due to inoculation of halotolerant microbial consortium under saline condition

**Figure 5:**
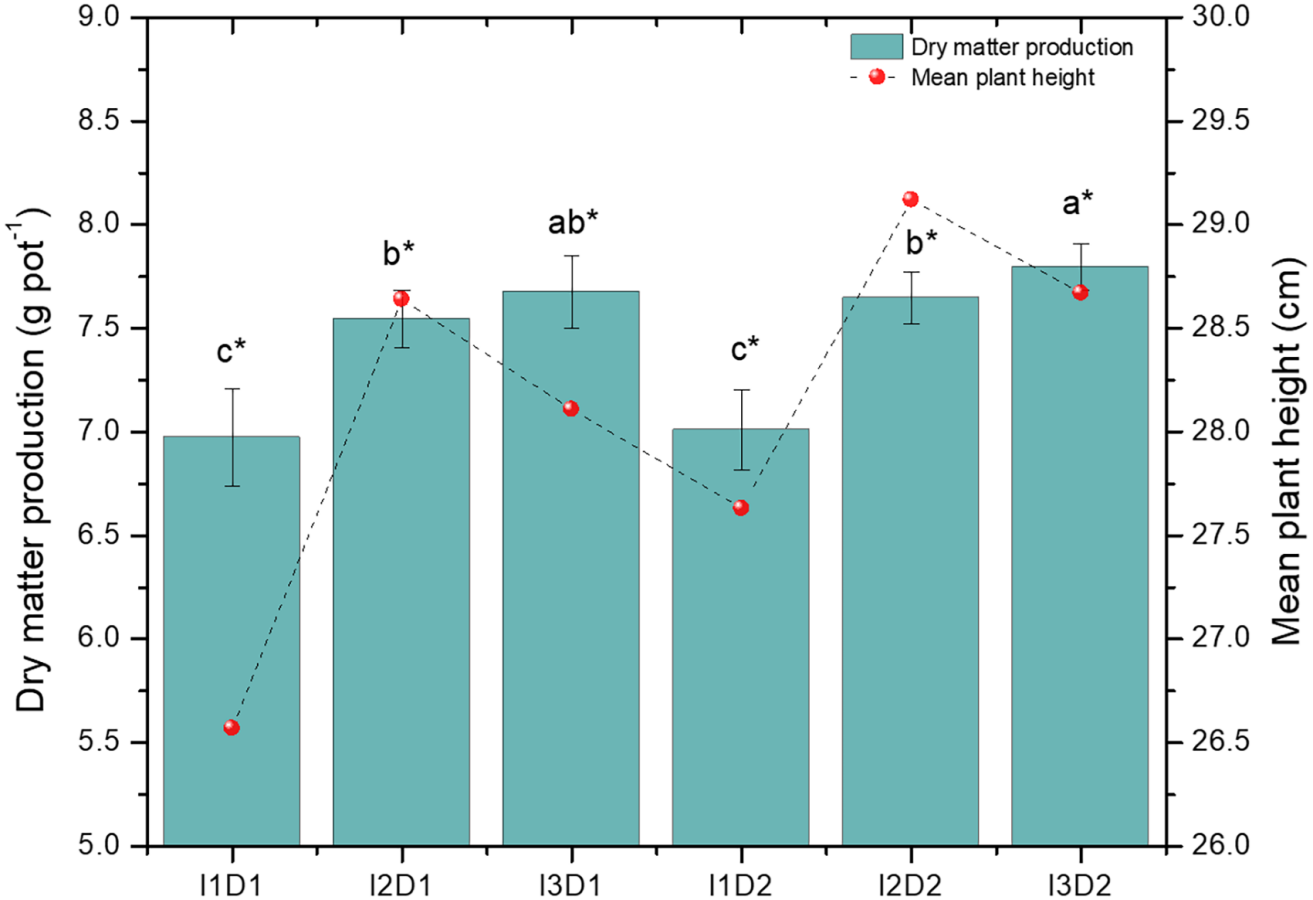
Effect of halotolerant microbial consortia dosage on growth of *Vigna mungo* L. under salinity. I_1_ – Control; I_2_ – Microbial consortium I; I_3_ – Microbial consortium II and D_1_-2 kg of bioformulation per ha; D_2_-4 kg bioformulation per ha. Bars that do not share similar letters denote statistical significance. (*denote significance at *p* < 0.05).

Legume crops are sensitive to soil salinity, as the external NaCl salinity rises, their ability to prevent Na from entering the photosynthetically active leaves decreases rapidly (Arora et al., 2020). In the present study, all the growth traits increased significantly in the microbial consortium applied treatments than the control. The root biomass and shoot biomass of *Vigna mungo* L. were found to be the highest in the treatment I_3_D_2_ with the values of 9.09 and 23.39 g pot^−1^, respectively (Figure 5). The least values for both the root (8.14 g pot^−1^) and shoot biomass (20.92 g pot^−1^) were recorded in I_1_D_1_. The presence of NaCl leads to specific ion toxicity that reduces the fresh and dry weight of control plants and is linked to reduced photosynthetic rate (Ambede et al., 2012). The plant height (29.10 cm) and dry matter production (7.80 g pot^−1^) were the highest in I_2_D_2_ and I_3_D_2_, respectively. The mean values of the plant height in the treatments I_1_, I_2_, and I_3_ were noted as 27.1, 28.9, and 28.4 cm, respectively. Growth attributes were significantly increased by the inoculation of microbial consortia. This increased shoot and root growth of inoculated plants could be attributed to phytohormone synthesis and bacterial N_2_ fixation, which led to increased water and nutrient uptake (Shukla et al., 2012). The promotion of shoot length, root length and germination percentage under salinity were evident in the *in vivo* studies, where, *B. velezensis, S. marcescens, K. radicincitans, B. niacini, M. indicus*, and *K. rhizophila* had significantly higher potential compared to other strains. Owing to this, the microbial consortia with these strains could have resulted in better growth attributes than uninoculated control. Similarly, halotolerant PGPR consortia with *Pseudomonas fluorescens* and *Acinetobacter* sp. exhibited a substantial increase in plant height by 15.98 and 26.82 per cent, respectively, than uninoculated control (Yasin et al., 2018). The MC-II (*B. velezensis*, *K. radicincitans* and *K. rhizophila*) inoculated at high dosage (4 kg ha^−1^) had significantly higher dry matter production (11.3%) and mean plant height (4%) than the control (Figure 5). The high ammonia and IAA synthesis by MC-II have resulted in increment of the growth rate of *Vigna mungo* L. even under saline conditions. Significant reduction in shoot biomass of control plants due to retardation of plant growth in uninoculated treatments was also observed in earlier studies (Rahman et al., 2017; Borlu et al., 2018; Hassan et al., 2018). The ability *of B. velezensis* and *K. rhizophila* with high ACC deaminase activity may have supported root growth by lowering the ethylene level in plants under saline conditions.

#### 3.9.3. Physiological parameters of *Vigna mungo* L.

**Figure 6:**
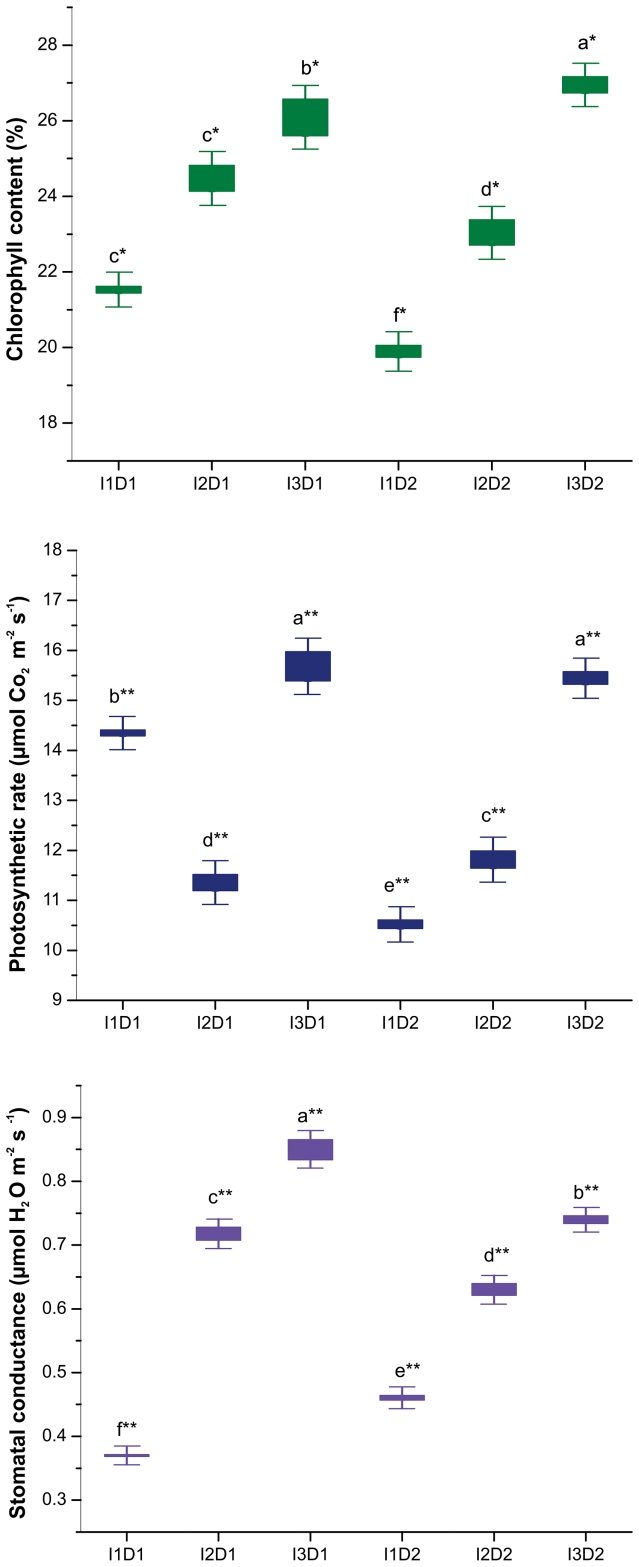
Effect of halotolerant microbial consortia dosage on physiological attributes of *Vigna mungo* L. under salinity. I_1_ – Control; I_2_ – Microbial consortium I; I_3_ – Microbial consortium II and D_1_-2 kg of bioformulation per ha; D_2_-4 kg bioformulation per ha. Bars that do not share similar letters denote statistical significance. (* and ** denote significance at *p* < 0.05 and *p* < 0.01, respectively).

The physiological parameters such as chlorophyll content, photosynthetic rate and stomatal conductance were found to be significantly higher in the MC inoculated treatments than the control (Figure 6). Treatment I_3_D_2_ followed by I_3_D_1_ had higher chlorophyll content of 26.95 and 26.10%, respectively compared to that of the control (I_1_D_1_ and I_1_D_2_). Subsequently, the lowest chlorophyll content of 19.88% was recorded in I_1_D_2_. Our findings are consistent with studies of Vimal and Singh (2019), who reported that PGPR inoculation increased chlorophyll content even under salinity stress. The prevention of nucleic acid damage in the chloroplast through reduction in ROS production by the inoculation of microbial consortium has led to increase in chlorophyll content in the active leaves. This in turn reflected in higher photosynthetic rate, since the amount of chlorophyll in a plant is directly proportional to its photosynthetic activity (Sapre et al., 2022). Treatment I_3_D_1_ followed by I_3_D_2_ had higher photosynthetic rate of 15.69 and 15.45 μmol CO_2_ m^−2^ s^−1^, respectively, compared to that of the control (I_1_D_1_ and I_1_D_2_). The least photosynthetic rate of 10.51 μmol CO_2_ m^−2^ s^−1^ was recorded in I_1_D_2_. PGPR isolates with ACC deaminase activity (*M. indicus, S. marcescens, B. velezensis, K. radicincitans*, and *K. rhizophila*) has been found to prevent reduction in chlorophyll content, and there by maintaining the photosynthetic rate of plants under saline stress (Habib et al., 2016). However, treatment I_3_D_1_ followed by I_3_D_2_ had higher stomatal conductance with 0.85 and 0.74 μmol H_2_O m^−2^ s^−1^, respectively, compared to that of the control (I_1_D_1_ and I_1_D_2_). The higher stomatal conductance in active leaves is an adaptive mechanism of plants to overcome the salt stress. This could be made possible by maintaining the turgor pressure in the guard cells. The siderophore production by the halotolerant PGPR increases the iron and potassium availability which are key nutrients in photosynthetic activity (Chauhan et al., 2022). Owing to this the sodium and potassium balance is attained in the plants. The inoculation of MC- II at 2 kg ha^−1^ (*B. velezensis, K. radicincitans*, and *K. rhizophila*) increased photosynthetic rate and stomatal conductance by 0.3 and 1.3 times, respectively, as compared to the control. This was supported by the research conducted by Bayuelo-Jimenez et al. (2012), who found that neither stomatal conductance nor photosynthetic activity was affected by salt stress due to high IAA production when inoculated with salt tolerant PGPRs.

#### 3.9.4. Yield attributes of *Vigna mungo* L.

**Figure 7:**
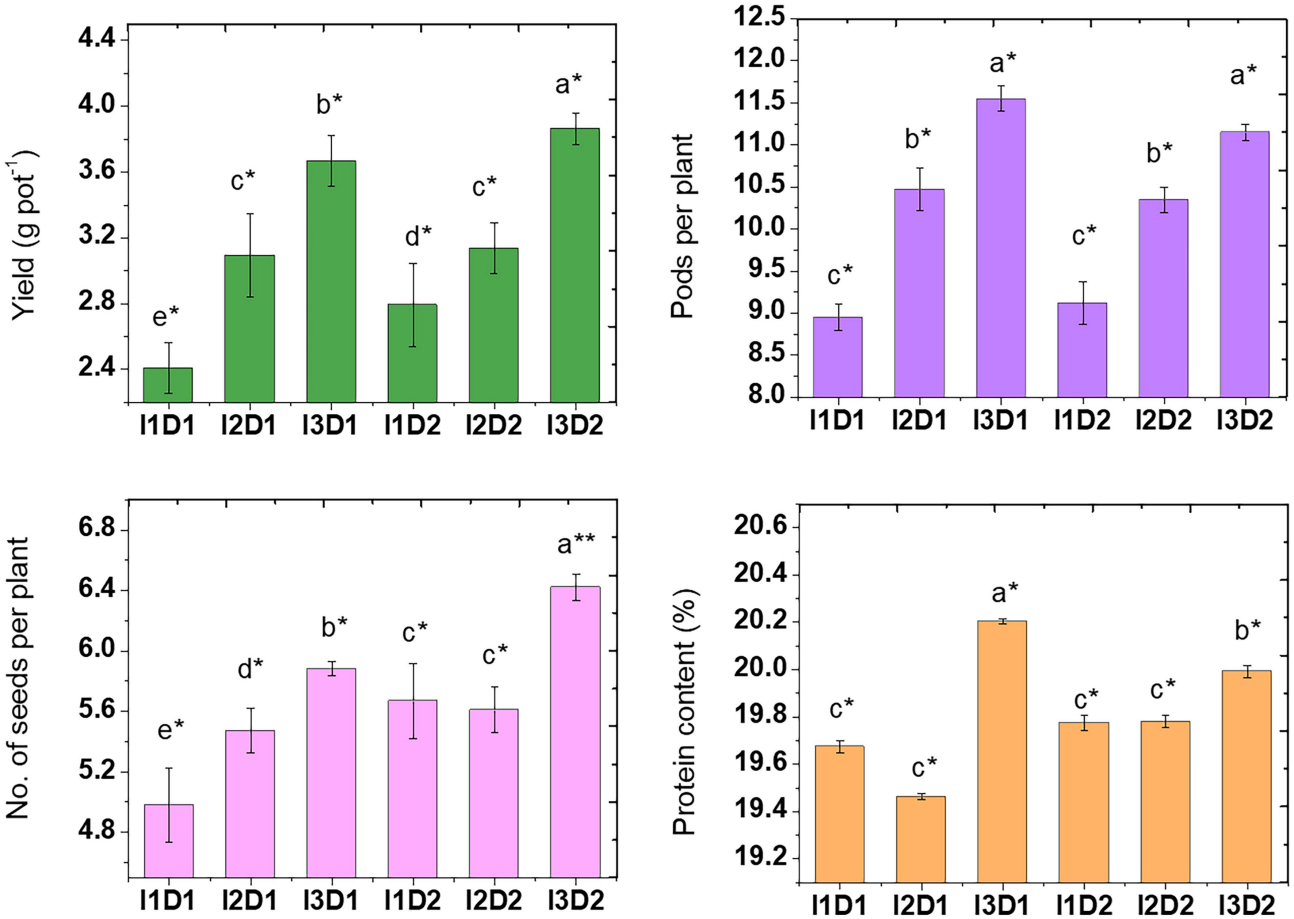
Effect of halotolerant microbial consortia dosage on yield quantity and quality of *Vigna mungo* L. under salinity. I_1_ – Control; I_2_ – Microbial consortium I; I_3_ – Microbial consortium II and D_1_-2 kg of bioformulation per ha; D_2_-4 kg bioformulation per ha. Bars that do not share similar letters denote statistical significance. (* and ** denote significance at *p* < 0.05 and *p* < 0.01, respectively).

The negative effects of salinity due to EC in soil (2.62 dS m^−1^) and irrigation water (2.5 dS m^−1^) have resulted in reduction of yield and protein quality in *Vigna mungo* L. The plant yield (3.87 g pot^−1^) and pods per plant (11.55) were highest in I_3_D_2_ and I_3_D_1_, respectively. The treatments applied with MC-II had a significantly higher number of pods per plant (25.5%) and grain yield (45%) than the control (Figure 7). Similar results were obtained by Saravanakumar and Samiyappan (2007), where pods count and protein content in peanut crop was increased upon inoculation with *Pseudomonas fluorescens* under saline stress. The mean values of the yield in the treatments I_1_, I_2_, and I_3_ were 2.60, 3.11, and 3.77 g pot^−1^, respectively. This indicates a significant improvement in crop yield due to microbial consortium inoculation that alters the nutritional environment through production of plant growth regulators (Patel et al., 2021). Among the treatments inoculated with microbial consortium, MC-II inoculated (*B. velezensis, K. radicincitans*, and *K. rhizophila*) had higher yield than MC-I (*M. indicus, S. marcescens*, and *N. Niacini*) inoculated. This is a direct indicator of high PGP potential of MC-II in comparison to MC-I.

Similarly, high protein content was recorded in I_3_D_1_ and I_3_D_2_ (MC-II inoculated treatments) with the corresponding values of 20.20 and 19.99%, respectively. Protein content in the seeds was significantly higher when inoculated with MC-II. The presence of more growth-promoting biochemicals in PGPR aids in the reduction of impacts caused by salt stress (Nadeem et al., 2016). Kavita and Alka (2010) stated that saline stress reduces N partitioning and fixation leading to lower protein accumulation in the seeds of legume plants. In line with this, seeds from the control treatment had low protein than microbial consortium inoculated treatments. The microbial consortia application improves nitrogen use efficiency, which in turn favored the accumulation of protein in seeds. Similarly, compared to the un-inoculated control, co-inoculation of *Bacillus* sp. and *Arthrobacter* sp. significantly increased protein content in the maize even under salt stress (100 mM NaCl; Hassan and Bano, 2015). The inoculation of PGPR with ACC deaminase activity on maize showed 3.3 folds increase in root length at EC 9 dS m^−1^ and shoot lengths increased by 2.3 folds over uninoculated control (Kausar and Shahzad, 2006). Inoculation of maize with salt tolerant PGPR and addition of organic amendments like poultry manure, cow dung and spent mushroom substrate has improved crop growth and fertility status of the soil in saline ecosystem. Further, the inoculation also reduced the sodium uptake by the crop thereby reducing the effect of salinity (Upadhyay and Chauhan, 2022).

### 3.10. Mechanism behind the improvement in crop growth through halotolerant PGPR inoculation and way forward

**Figure 8:**
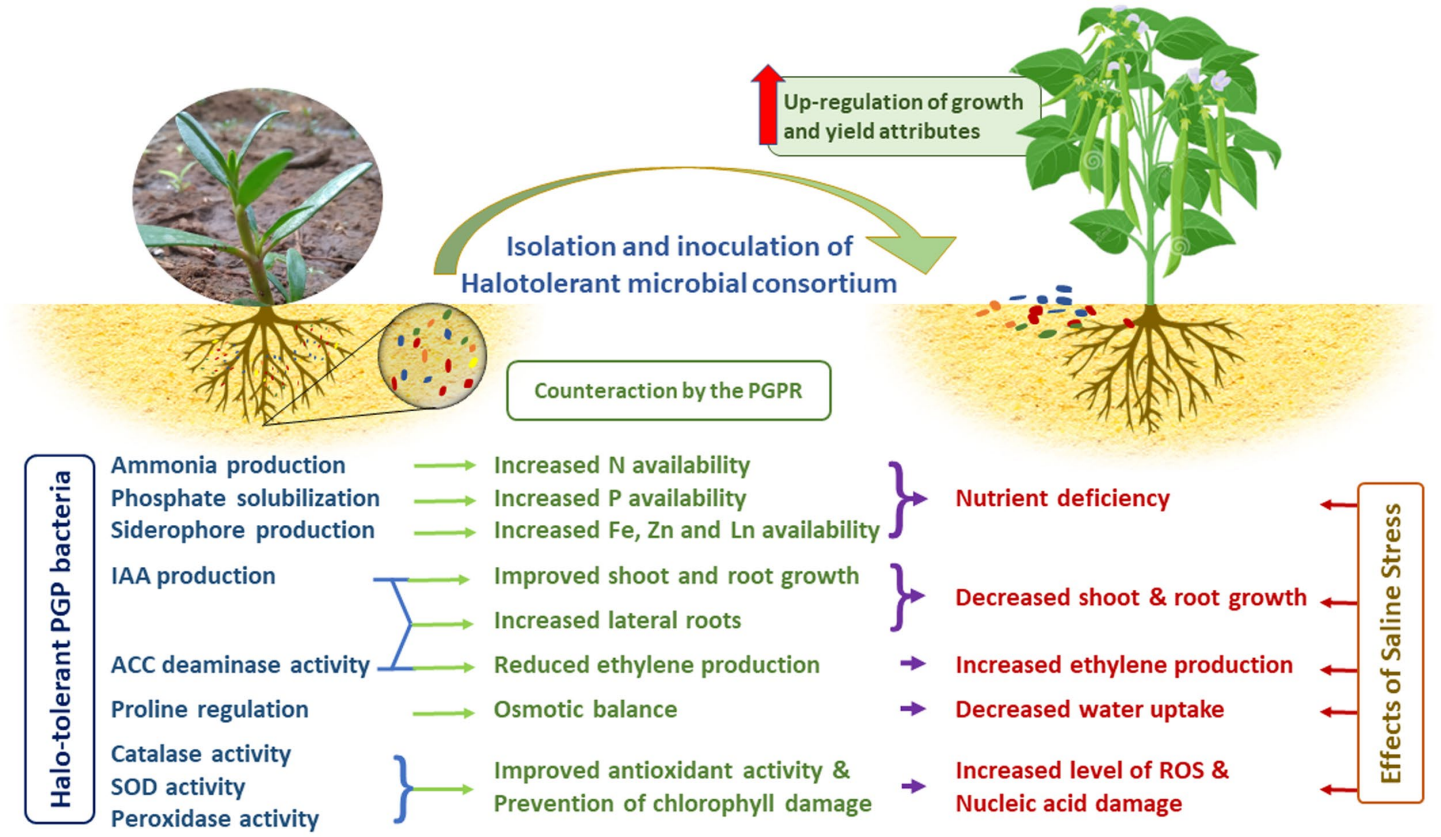
Prospects of halotolerant microbial consortia for alleviation of saline stress.

Several reports have shown that halotolerant PGPRs effectively improve the growth of various agricultural crops under salinity stress conditions (Qin et al., 2016; Singh and Jha, 2016a; Etesami and Noori, 2019; Orhan and Demirci, 2020). Mechanisms by which they impact growth are (i) triggering plant antioxidant defense by upregulating vital enzymes that scavenge excess ROS, (ii) alleviating nutrient deficiency by fixing atmospheric nitrogen and producing ammonia, solubilizing P, producing siderophores for Fe, Zn and Ln uptake, (iii) increasing the uptake of selected ions for maintaining a high K^+^/Na^+^ ratio (John and Lakshmanan, 2018), (iv) decreasing plant Na^+^ accumulation by excreting exopolysaccharide to bind cations (especially Na^+^) in roots and prevent their translocation to leaves, and (v) reduction in ROS production by counteracting ethylene synthesis (Figure 8). Probably, different mechanisms are involved in each interaction that leads to plant growth stimulation under saline conditions. The prevention of nucleic acid and chlorophyll damage by quenching the ROS plays a major role in improving the salt tolerance in the crops. Nevertheless, the variation in chloroplast structure and SOD activity among the different plants interferes with the PGPR ability in stress alleviation. Hence, a bacterial strain that stimulates the growth of a particular plant species in the presence of salt may not cause similar effect in other plants. Furthermore, many intricate mechanisms are involved when endophytes come into the picture (Santoyo et al., 2016; Khan et al., 2017). The endophyte, *Burkholderia phytofirmans* enhanced the growth of six switchgrass cultivars out of the eight that were tested (Khan et al., 2017). Inoculation with this strain was found to induce wide-spread changes in gene expression in the host plant, including transcription factors that are known to regulate the expression of some plant stress factor genes (Lara-Chavez et al., 2015). It is likely that changes in plant gene expression could also be induced by the halotolerant consortium used in this study to inoculate *Vigna mungo* L. Hence further investigation of the genomic interaction with the plants is in process. This would have a significant influence in identifying microbial inoculums that guarantees the successful cultivation of crops under variety of stressful environments.

## 4. Conclusion

The current study revealed the plant growth-promoting attributes of halotolerant PGPR on *Vigna mungo* L., grown under saline conditions. Among the nine isolates, *S. marcescens, B. velezensis, K. rhizophila*, and *K. radicincitans* improved germination percentage, vigor index, shoot and root length of *Vigna mungo* L. In the pot study, *Vigna mungo* L. inoculated with microbial consortia (*K. rhizophila, B. velezensis*, and *K. radicincitanas* – MC-II) @ 4 kg ha^−1^ exhibited higher concentration of chlorophyll, photosynthetic rate and stomatal conductance. The poor leaf osmolyte (proline) content and antioxidant enzymes (catalase and superoxide dismutase) activities indicated lesser salt stress in inoculated plants over uninoculated control. Consequently, significant increase in growth and yield of *Vigna mungo* L. in soils salinized by long term irrigation of paper and pulp mill effluent was observed. Therefore, halotolerant microbial consortia, MC-II having the potential to induce salt stress tolerance can enhance crop growth. However, further research must be oriented towards improving the host plant-microbial compatibility to increase the salt tolerance and productivity of wide variety of crops. Utilization of biostimulants, nano-particles and bio-amendments along with PGPR is another emerging area of research which has the potential to improve the crop microbe association under saline conditions.

## Data availability statement

The original contributions presented in the study are included in the article/Supplementary material, further inquiries can be directed to the corresponding authors.

## Author contributions

JJ has conducted all the experiments under the guidance of MM and TK. JJ and CP completed the formal analysis and the first draft of the manuscript. BG, SSR, and SR participated in the analysis of experimental results. MP, EK, and VD directed the manuscript writing and revision of the manuscript. All authors have read and agreed on the final version of the text.

## Funding

The financial assistance to carry out the research was provided by Tamil Nadu Newsprint and Papers Limited, Karur, India.

## Conflict of interest

The authors declare that the research was conducted in the absence of any commercial or financial relationships that could be construed as a potential conflict of interest.

## Publisher’s note

All claims expressed in this article are solely those of the authors and do not necessarily represent those of their affiliated organizations, or those of the publisher, the editors and the reviewers. Any product that may be evaluated in this article, or claim that may be made by its manufacturer, is not guaranteed or endorsed by the publisher.
