# Supplementary material for: Biomining Sesuvium portulacastrum for halotolerant PGPR and endophytes for promotion of salt tolerance in Vigna mungo L
Source: Front Microbiol. 2023 Feb 14;14:1085787. doi: 10.3389/fmicb.2023.1085787 (PMC9971939; doi:10.3389/fmicb.2023.1085787)
Supplement: Supplementary file 1 [file Data_Sheet_1.docx]

**Supplementary Table S1. Composition of the Nutrient Agar medium**

| **S.No.** | **Particulars** | **Quantity** |
| --- | --- | --- |
| **1.** | Peptone | 5.0 g |
| **2.** | Beef extract | 3.0 g |
| **3.** | NaCl | 5.0 g |
| **4.** | Distilled water | 1000 ml |
| **5.** | Agar | 20.0 g |

***Note:*** *pH should be maintained at 7.0 for solidification*

**Supplementary Table S2. Morphological and biochemical characters of the halotolerant microbial isolates**

| **Strains** | **Gram reaction** | **Cell morphology** | **Motility** | **Casein** | **Starch** | **Citrate** | **Amylase** | **Catalase** | **Oxidase** | **Urease** | **Indole** | **MR-VP** | **Nitrate** | **Protease** |
| --- | --- | --- | --- | --- | --- | --- | --- | --- | --- | --- | --- | --- | --- | --- |
| **SPP 2** | Positive | Single celled and rod shaped | Non-motile | **+** | **+** | **-** | **+** | **+** | **+** | **-** | **+** | **-** | **-** | **+** |
| **SPP 5** | Positive | Single celled and rod shaped | Non-motile | **-** | **-** | **+** | **+** | **+** | **+** | **-** | **-** | **-** | **+** | **+** |
| **SPP 6** | Negative | Single celled and rod shaped | Non-motile | **-** | **+** | **+** | **+** | **+** | **-** | **+** | **-** | **+** | **-** | **+** |
| **SPTT 3** | Positive | Single celled and rod shaped | Motile | **+** | **+** | **+** | **+** | **+** | **+** | **-** | **-** | **+** | **+** | **+** |
| **SPTT 7** | Positive | Single celled short rods | Motile | **+** | **-** | **+** | **+** | **+** | **-** | **-** | **-** | **+** | **+** | **+** |
| **SPTT 8** | Positive | Single and coccoid cells | Non-motile | **+** | **-** | **-** | **+** | **+** | **+** | **+** | **-** | **+** | **-** | **+** |
| **SPTV 3** | Positive | Single celled and rod shaped | Non-motile | **-** | **+** | **+** | **+** | **+** | **-** | **-** | **-** | **+** | **-** | **+** |
| **SPTVE 3** | Negative | Single celled and rod shaped | Motile | **+** | **-** | **-** | **+** | **+** | **-** | **+** | **-** | **-** | **+** | **+** |
| **SPTVE 4** | Negative | Single celled and rod shaped | Motile | **-** | **+** | **+** | **+** | **+** | **-** | **-** | **-** | **+** | **-** | **+** |

**Supplementary Table S3. Physicochemical properties of the experimental soil in pot culture**

| **S. No** | **Parameter** | **Values** |
| --- | --- | --- |
|  | pH | 8.18 |
|  | EC (dS m^-1^) | 2.62 |
|  | Organic carbon (%) | 0.63 |
|  | Available nitrogen (kg ha^-1^) | 241 |
|  | Available phosphorus (kg ha^-1^) | 17.64 |
|  | Available potassium (kg ha^-1^) | 297 |
|  | Exchangeable calcium (cmol (p^+^) kg^-1^) | 8.89 |
|  | Exchangeable magnesium (cmol (p^+^) kg^-1^) | 2.90 |
|  | Exchangeable sodium (cmol (p^+^) kg^-1^) | 1.84 |
|  | Exchangeable potassium (cmol (p^+^) kg^-1^) | 0.52 |
|  | Exchangeable chloride (cmol (p^-1^) kg^-1^) | 0.25 |
|  | Cation exchange capacity (cmol (p^-1^) kg^-1^) | 14.17 |
|  | ESP (%) | 13.54 |

**Supplementary Figure 1. Phosphate solubilization by the halotolerant isolates in Pikovskayas medium**

**Supplementary Figure 2. Siderophore production by the halotolerant isolates in CAS blue agar plate**
